# Boosting the Sodiation Kinetics of Sn Anode Using a Yolk–Shell Nanohybrid Structure for High‐Rate and Ultrastable Sodium‐Ion Batteries

**DOI:** 10.1002/advs.202408450

**Published:** 2024-10-31

**Authors:** Hyojun Lim, Seungho Yu, Wonyoung Chang, Kyung Yoon Chung, Wonchang Choi, Sang‐Ok Kim

**Affiliations:** ^1^ Energy Storage Research Center Korea Institute of Science and Technology (KIST) 5, Hwarang‐ro 14‐gil, Seongbuk‐gu Seoul 02792 Republic of Korea; ^2^ Division of Energy & Environment Technology, KIST School Korea University of Science and Technology 5, Hwarang‐ro 14‐gil, Seongbuk‐gu Seoul 02792 Republic of Korea; ^3^ Department of Nuclear Science and Engineering and Department of Materials Science and Engineering Massachusetts Institute of Technology Cambridge MA 02139 USA; ^4^ Department of Energy Engineering Konkuk University 120 Neungdong‐ro, Gwangjin‐gu Seoul 05029 Republic of Korea

**Keywords:** hierarchical yolk–shell nanohybrid, high‐capacity anodes, Na‐ion batteries, sodiation kinetics

## Abstract

Metallic Sn (Tin) is a promising anode material for Na‐ion batteries owing to its high theoretical capacity of 870 mAh g^−1^. However, its large volumetric changes, interfacial instability, and sluggish sodiation kinetics limit its practical applications. Herein, a hierarchical yolk–shell nanohybrid composed of an Sn yolk and a Carbon/Silicon oxycarbide (C/SiOC) bilayer shell is prepared via the simple pyrolysis of a silicone oil dispersion containing an Sn precursor. The multifunctional bilayer helps boost sodiation kinetics by providing conductive pathways, enhancing the reversible capacity through surface capacitive reactions, and stabilizing the electrode/electrolyte interface. Abundant void interspaces inside the yolk–shell structure accommodate large volume changes of the Sn yolk. The Sn@C/SiOC nanohybrid demonstrates high specific capacity (≈500 mAh g^−1^ at 1 A g^−1^), remarkable rate performance up to 10 A g^−1^, and ultrastable cyclability (91.1% retention after 1500 cycles at 5 A g^−1^). This yolk–shell nanohybrid structuring can guide the development of various high‐capacity anodes for energy storage applications.

## Graphical abstract

A yolk–shell structured Sn@C/SiOC nanohybrid is designed to overcome sluggish sodiation and structural deterioration of Sn anode upon cycling. Strategic combination of unique heterostructure and mesoporous/robust shell boosts sodium‐ion diffusion kinetics through efficient charge transport channels and relieves mechanical stress induced by a large volume change, significantly improving the cycling stability and rate capability.

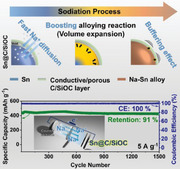

## 1 Introduction

Alloying‐type (i.e., Sn, Sb, and P) materials have gained significant attention as high‐capacity anodes for sodium‐ion batteries (SIBs).^[^ ^1^, ^2^, ^3^, ^4^, ^5^ ^]^ Particularly, metallic Sn has been considered owing to its abundance on earth and high theoretical capacity of 870 mAh g^−1^.^[^ ^6^, ^7^ ^]^ Nevertheless, Sn anodes exhibit restricted cycling stability from massive volume variation of ≈420% and particle aggregation due to alloying and dealloying reactions with sodium.^[^ ^8^ ^]^ Additionally, the sodiation kinetics of Sn is substantially sluggish owing to the formation of various intermediate phases,^[^ ^9^, ^10^ ^]^ further lowering the reversible capacity and degrading the performance; therefore, versatile strategies have been proposed.^[^ ^4^, ^11^, ^12^, ^13^, ^14^ ^]^ Various well‐controlled structures (nano‐, hollow‐, and yolk–shell structures) can facilitate the transfer of electrons and ions and alleviate mechanical strains in large volume variations of alloying anodes, enhancing structural durability and electrical/ionic conductivity upon repeated battery cycling.^[^ ^15^, ^16^, ^17^, ^18^ ^]^ The formation of heterostructures comprising metal‐semiconductor or semiconductor–semiconductor junctions with different work functions has improved the charge transport capability through the built‐in electric field at the hetero‐interface.^[^ ^19^, ^20^, ^21^, ^22^ ^]^ The adoption of carbonaceous materials as conductive buffer frameworks effectively suppresses drastic volume variations of host materials and offers a high electrical conductivity of active materials, demonstrating an improved sodium storage performance.^[^ ^12^, ^16^, ^23^, ^24^, ^25^ ^]^ Despite the benefits of the carbonaceous matrix, insufficient mechanical robustness and elasticity remain challenges to ensuring the structural reinforcement and enhanced cycling stability of electrode materials.

Silicon oxycarbide (SiOC) has been used as an effective buffer phase to mitigate the large volume changes of high‐capacity anode materials owing to its elastic and robust nature and superior electrical conductivity compared to that of carbon‐based materials. It has also been used as an active material in lithium‐ion batteries based on its moderate electrochemical reactivity with lithium and a reversible capacity of ≈500 mAh g^−1^.^[^ ^26^, ^27^, ^28^ ^]^ Although the conventional synthetic methods for SiOC ceramic materials are complicated and/or expensive, a cost‐effective approach through a simple, facile pyrolysis of an affordable silicone oil (SO) precursor has been reported.^[^ ^29^, ^30^, ^31^ ^]^ Similar to pure SiOC, several SiOC‐based hybrid materials, such as Si‐embedded in SiOC matrix composites and core–shell structured MoS_2_@SiOC, can be readily realized by this technique, and improved lithium‐ and sodium‐ion storage characteristics have been demonstrated.^[^ ^32^, ^33^ ^]^ The enhanced electrochemical performance of the SiOC‐containing high‐capacity anode materials is attributed to their excellent mechanical/chemical stabilities, good volume accommodation effects, and additional charge storage capability.^[^ ^29^, ^31^ ^]^ However, optimization of the proportion, structure, and morphology of SiOC in hybrid materials is paramount, as these properties significantly affect the overall reversible capacity and cycle/rate performance.

To overcome the sluggish sodiation issues and massive volume variation of Sn, we propose an efficient, synthetic, and hierarchically designed nanohybrid consisting of a Sn yolk and C/SiOC bilayer shell, denoted as Sn@C/SiOC. A spherical‐shaped, submicrometer‐sized Sn@C/SiOC nanohybrid was synthesized via a simple two‐step pyrolysis of SO suspensions of hollow‐SnO_2_@polydopamine (h‐SnO_2_@PDA). The metallic Sn yolk generated after the thermal reduction process was uniformly encapsulated within the multifunctional C/SiOC bilayer, with a void interspace that alleviates the large volume change of Sn yolk during redox reactions. Additionally, the robust C/SiOC outer layer in the nanohybrid structure served as a volumetric buffer matrix, further reinforcing the structural integrity. Its mesoporous characteristics enabled fast sodium‐ion diffusion kinetics upon sodiation and additional surface capacitive sodium‐ion storage. The Sn@C/SiOC demonstrated outstanding sodium storage performance in terms of a high reversible capacity, an ultrastable cycling performance, superior rate capability, and stable full‐cell performance, making it a promising anode candidate for advanced SIBs.

## 2 Results and Discussion

### 2.1 Design and Synthesis of the Sn@C/SiOC Nanohybrid

**Figure 1:**
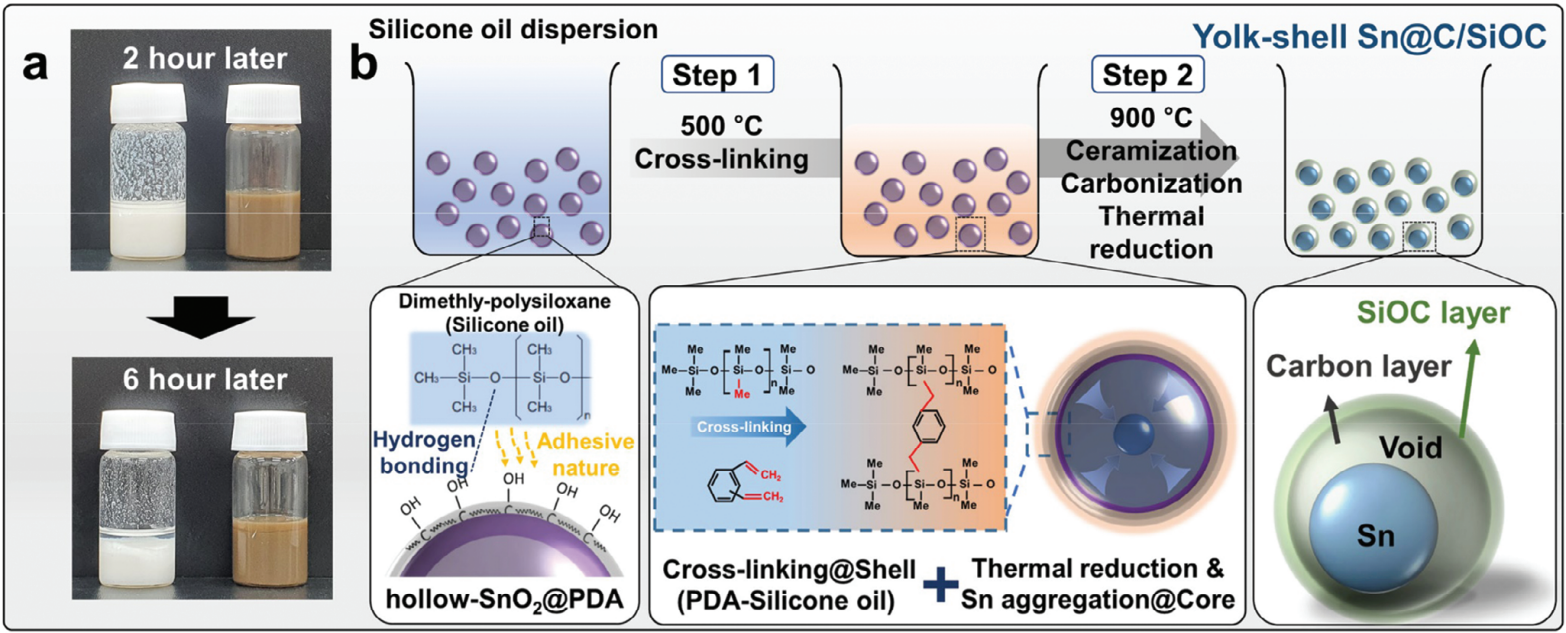
a) Digital photographs of the SO suspension containing SnO_2_‐based precursors (left: h‐SnO_2_ and right: h‐SnO_2_@PDA) and b) schematic of the preparation processes of the Sn@C/SiOC.

The digital photographs of two different SO suspensions of h‐SnO_2_ (white) and h‐SnO_2_@PDA (brown) precursors are presented in **Figure** **1**a. These precursors were homogeneously dispersed in SO, and both suspensions were stored for up to 6 h without magnetic stirring to compare the changes in the dispersion characteristics over time. Due to the high viscosity of SO, both samples preserved a uniform dispersion of h‐SnO_2_‐based particles for 2 h. The h‐SnO_2_ suspension revealed a prominent sedimentation after 6 h; in contrast, the h‐SnO_2_@PDA suspension retained its initially well‐dispersed state, indicating a significantly enhanced stability. Based on the Fourier transform‐infrared spectra, only the h‐SnO_2_@PDA shows the presence of organic functional groups (i.e., C*─*O, C═C, N*─*H, *─*OH, and *─*NH), which are from the PDA coating layer (Figure S1, Supporting Information). The *─*OH functional group in the PDA layer can form hydrogen bonds with oxygen species in the SO, which stabilizes the dispersion and allows the formation of a SO‐derived uniform shell in Sn@C/SiOC end products.

Figure 1b shows the synthesis of the Sn‐based anode materials with a hierarchical yolk–shell nanohybrid structure composed of Sn nanoparticles within a nanoscale C/SiOC bilayer. According to a previous study, the h‐SnO_2_@PDA precursor was synthesized hydrothermally using the self‐polymerization reaction of dopamine hydrochloride.^[^ ^15^, ^34^ ^]^ The self‐polymerization reaction of dopamine hydrochloride under the alkaline solution and the in situ coating of the PDA generated the h‐SnO_2_ precursor with a uniform PDA coating layer. In addition, the as‐prepared h‐SnO_2_@PDA precursor was completely dispersed in SO through ultrasonication and magnetic stirring. The SO can physically or chemically adhere to the precursor surface owing to the adhesive properties of PDA and hydrogen bonds and the viscous nature of SO. Subsequently, the continuous two‐step heat treatment of the SO suspension was conducted in a quartz tube furnace under an argon atmosphere. In the first pyrolysis step at 500 °C for 4 h, the SO located at the surface of h‐SnO_2_@PDA was transformed into a thin, uniform preceramic coating layer via cross‐linking reactions between polysiloxane‐related groups in SO and phenyl‐related groups in divinylbenzene at temperatures greater than 400 °C. The excess polysiloxane group that did not participate in the cross‐linking reaction was completely removed through the depolymerization reaction of SO, owing to its highly volatile characteristics.^[^ ^30^, ^35^, ^36^ ^]^ In the second pyrolysis step with a temperature of 900 °C conducted for 1 h, the hierarchical yolk–shell structured Sn‐based nanohybrid with a nanoscale C/SiOC bilayer was fabricated via simultaneous reactions including the carbothermal reduction of SnO_2_ to Sn supported by the PDA coating layer as a reduction agent, ceramization of preceramic into SiOC, and carbonization of PDA. Note that the metallic Sn particles generated from h‐SnO_2_ during the carbothermal reduction process subsequently aggregated into metallic Sn yolk clusters in the C/SiOC shell with a void interspace derived from the PDA backbone and SO.

### 2.2 Morphology and Structure

**Figure 2:**
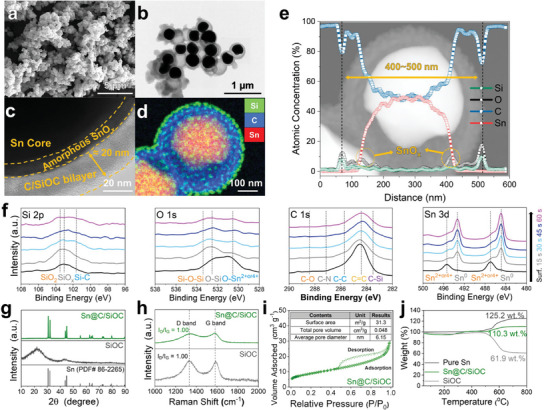
a) FE‐SEM, b) TEM, and c) HR‐TEM images of Sn@C/SiOC. d) EDS mapping image (Si, O, and Sn) and e) corresponding cross‐sectional line scan profile (Si, O, C, and Sn) of the Sn@C/SiOC nanohybrid particle. f) XPS depth profiles of Sn@C/SiOC in the regions of Si 2p, O 1s, C 1s, and Sn 3d. g) XRD patterns and h) Raman spectra of SiOC and Sn@C/SiOC. i) Nitrogen adsorption–desorption isotherm and surface information (inset) of Sn@C/SiOC. j) TGA curves of pure Sn, SiOC, and Sn@C/SiOC.

Particle morphology and the detailed microstructure of the Sn@C/SiOC samples were observed by performing combined microscopic analyses. The h‐SnO_2_@PDA precursor shows submicroscale core SnO_2_ particles (400–500 nm) covered with a nanoscale PDA coating layer of ≈20 nm (Figure S2, Supporting Information). After the two‐step heat treatment, images obtained from field emission‐scanning electron microscopy (FE‐SEM) and transmission electron microscopy (TEM) (**Figure** **2**a,b) exhibited spherical‐shaped, submicrometer‐sized (400–500 nm) particles composed of well‐confined Sn nanoparticles in partial contact with the C/SiOC bilayer shell along with small void interspaces. This demonstrates the formation of hierarchical yolk–shell structured Sn@C/SiOC. Compared to the SnO_2_@PDA precursor, no dominant changes in either shape or size were observed, implying that the generation of a bilayered C/SiOC shell from the SO occurred only at the outermost surface of the nanohybrid particles. Moreover, the Sn@C material was also prepared by heat treatment of the h‐SnO_2_@PDA precursor only, without the dispersion step, to further confirm the effects of the SO on the final size and morphology. The TEM image of the Sn@C hybrid (Figure S3, Supporting Information) reveals many empty carbon shells, with the formation of metallic Sn extracted from the carbon shell during pyrolysis aggregated because of the low melting point of Sn (231 °C).^[^ ^37^ ^]^ This implies that the carbon shell has insufficient mechanical strength to prevent the extrusion of metallic Sn, whereas the preceramic coating layer derived from SO preserves the presence of metallic Sn inside the core during the two‐step heat treatment. According to the images obtained by the high resolution‐TEM (HR‐TEM) equipped with energy dispersive X‐ray spectroscopy (EDS) and the corresponding cross‐sectional line scanning profile of the Sn@C/SiOC (Figure 2c–e), it can be confirmed that the nanoscale C/SiOC bilayer (≈20 nm) and the very thin amorphous SnO*_x_* layer (< 10 nm) are uniformly coated on the surface of the Sn yolk. The amorphous SnO*_x_* layer is believed to originate from the partial surface oxidation or chemical reaction between Sn and SO. Therefore, the hierarchically designed Sn@C/SiOC consisting of a robust C/SiOC bilayer and void space can effectively accommodate the massive volume change of the metallic Sn during the sodiation/desodiation processes because of the unique and robust yolk–shell heterostructure containing sufficient void spaces, resulting in enhanced long‐term cycling stability.

The X‐ray photoelectron spectroscopy (XPS) depth profile measurement was conducted for Sn@C/SiOC (Figure 2f) to elucidate the variation in the chemical bonding states from the shell to yolk regions. Prior to ion sputtering, the Si 2p binding energy spectra of Sn@C/SiOC can be divided into three prominent peaks: 101.6, 103.1, and 103.5 eV, corresponding to Si*─*C, SiO*_x_*, and SiO_2_ bonds, respectively.^[^ ^38^ ^]^ Moreover, the C 1s spectra can be deconvoluted into five peaks located at 283.5, 284.4, 285.7, 287.1, and 288.4 eV, corresponding to C*─*Si, C═C, C*─*C, C*─*N, and C*─*O bonds, respectively. This indicates the presence of the C/SiOC bilayer from the thermal decomposition of the PDA and SO precursors.^[^ ^29^, ^39^, ^40^ ^]^ Additionally, the O 1s spectra demonstrate the SiOC related peaks, including Si*─*O*─*Si (533.6 eV) and O*─*Si (532.7 eV).^[^ ^29^ ^]^ These results are consistent with previous studies on nitrogen‐doped carbon and SiOC‐based materials.^[^ ^29^, ^39^, ^40^ ^]^ During depth profiling, the XPS spectra in regions Si 2p, C 1s, and O 1s show a gradual decrease in the intensity of the peaks associated with the C/SiOC bilayer. Simultaneously, the Sn 3d spectra indicate the evolution and subsequent growth of the peaks related to metallic Sn over the sputtering time, demonstrating the hierarchical yolk–shell structure. Interestingly, the O 1s and Sn 3d spectra exhibited dominant peaks associated with Sn^2+^ and Sn^4+^ (≈487 and ≈495 eV, respectively) and O*─*Sn^2+ or 4+^ (≈530 eV) bonds, confirming that the thin SnO*_x_* layer was located at the surface of the Sn yolk.^[^ ^41^, ^42^, ^43^, ^44^, ^45^ ^]^

X‐ray diffraction (XRD), Raman spectroscopy, nitrogen adsorption–desorption analysis, and thermogravimetric analysis (TGA) were conducted to identify the structural and compositional characteristics (Figure 2g–j). Figure 2g compares the XRD patterns of SiOC and Sn@C/SiOC. In the former, two broad diffraction peaks at ≈23° and 44° were observed, corresponding to the amorphous silica or Si*─*O*─*C glass phase.^[^ ^29^ ^]^ The latter shows diffraction peaks corresponding to metallic Sn (PDF# 86‐2265) without any impurity phases, indicating that SnO_2_ in the SnO_2_@PDA precursor is completely converted into metallic Sn during heat treatment (Figure S4, Supporting Information). However, contrary to the HR‐TEM and XPS results, the characteristic peaks of SnO*_x_* were not detected by XRD, probably because of its amorphous nature. From the Raman spectra of SiOC and Sn@C/SiOC in the range of 1200–1700 cm^−1^ (Figure 2h), two prominent peaks were observed at 1330 and 1580 cm^−1^, which are attributed to the D‐band (disordered carbon: sp^3^‐hybridized vibration) and G‐band (graphitic carbon: in‐plane sp^2^‐stretching), respectively, whereas pure Sn exhibited no corresponding peaks.^[^ ^46^, ^47^ ^]^ The ratio of the intensities of the D‐ to G‐bands (*I*_D_/*I*_G_) indicates the degree of graphitic characteristics, which is an important factor in determining the electrical conductivity. As a lower degree of graphitization reveals a higher electrical conductivity,^[^ ^48^, ^49^ ^]^ the high *I*_D_/*I*_G_ value of ≈1.0 demonstrates that the nanohybrid material with a conductive C/SiOC bilayer ensures high conductivity, improved reaction kinetics, and high‐rate performance.

The nitrogen adsorption–desorption analysis was performed to investigate the Brunauer–Emmett–Teller (BET) surface area and the pore size distribution of Sn@C/SiOC, which are shown in Figure 2i and displayed as type‐IV adsorption–desorption isotherm curves, indicating mesoporous characteristics with pore diameters ranging from 2 to 50 nm. The BET surface area and total pore volume of Sn@C/SiOC were measured to be 31.3 m^2^ g^−1^ and 0.048 cm^3^ g^−1^, respectively. The high surface area and total pore volume of Sn@C/SiOC originate from the mesoporous characteristics of SiOC (Figure S5, Supporting Information). Therefore, Sn@C/SiOC with a mesoporous structure can provide fast ionic transportation channels and more abundant redox‐active sites for charge carriers, enabling superior high‐rate capability. Additionally, the amount of Sn and C/SiOC bilayer components in the hybrid material can be estimated from the TGA results, as shown in Figure 2j. The Sn, Sn@C/SiOC, and SiOC TGA curves showed negligible weight changes for temperatures up to 400 °C because of the removal of small amounts of adsorbed water or chemical residues on the particle surface. Above 400 °C, huge weight changes were observed mainly because of the oxidation of carbon (C (*s*) → CO_2_ (*g*)) and Sn (Sn (*s*) → SnO_2_ (*s*)). Based on the mass‐balance calculations using the TGA curves, Sn@C/SiOC is considered to contain ≈86.9 wt% of Sn and 13.1 wt% of the C/SiOC bilayer. Consequently, the high Sn content may compensate for the reduction in tap density caused by voids in the yolk–shell structure and enable the fabrication of Sn‐based anodes with high energy density, which is beneficial for practical applications.

### 2.3 Electrochemical Performance in SIBs

#### 2.3.1 Half‐Cell Test Results

**Figure 3:**
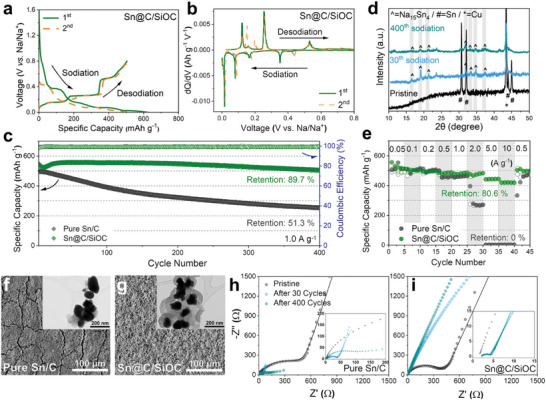
a) Galvanostatic charge–discharge voltage profiles for the 1st and 2nd cycles of the Sn@C/SiOC electrode measured at 0.05 A g^−1^ (≈0.07 C‐rate, 1 C‐rate for Sn@C/SiOC: –760 mAh g^−1^). b) d*Q*/d*V* curve from the data in (a). c) Cycling performance of the Sn‐based anodes at 1.0 A g^−1^ (≈1.3 C‐rate). d) Ex situ XRD patterns of the Sn@C/SiOC electrodes after various cycles. e) Rate performance of the Sn‐based anode materials at various current densities from 0.05 to 10 A g^−1^ (0.07–13 C‐rate). Top‐view SEM surface images of f) pure Sn/C and g) Sn@C/SiOC electrodes. The inset images in f) and g) are low‐magnification TEM images of pure Sn/C and Sn@C/SiOC particles after 400 cycles. Nyquist plots of h) pure Sn/C and i) Sn@C/SiOC electrodes obtained at various numbers of cycles (before cycling, after 30, and 400 cycles).

The sodium‐ion storage performance of the Sn‐based electrodes was investigated through various galvanostatic or potentiostatic electrochemical tests, which are summarized and compared in Table S1 (Supporting Information). The galvanostatic voltage profiles of the Sn@C/SiOC electrode for the initial two cycles measured at a current density of 0.05 A g^−1^ are presented in **Figure** **3**a. With an initial Coulombic efficiency (CE) of 80.7%, the Sn@C/SiOC electrode exhibited discharge and charge capacities of 624.6 and 504.0 mAh g^−1^, respectively. Furthermore, the initial discharge/charge capacities and corresponding CE of the Sn/C electrode were 615.0/466.1 mAh g^−1^ and 75.8%, respectively (Figure S6a, Supporting Information). The higher initial specific capacities of the Sn@C/SiOC electrode compared to the Sn/C are attributed to the C/SiOC bilayer's surface capacitive charge storage capability.^[^ ^31^, ^50^ ^]^ Interestingly, both Sn‐based electrodes exhibited relatively higher initial CEs (> 75%) compared to previous literature on Sn‐based anodes for SIBs.^[^ ^3^, ^49^ ^]^ Generally, Sn‐based anodes show relatively low initial CE values (< 70%) in carbonate‐based electrolytes, resulting from the excessive formation of solid electrolyte interphase (SEI) and a sluggish activation process.^[^ ^1^, ^3^, ^4^, ^51^, ^52^, ^53^ ^]^ Here, according to a previous study, the use of ether‐based electrolytes instead of carbonate‐based ones may contribute to the improvement of the initial CE through the formation of a more stable and thinner SEI layer with enhanced ionic diffusivity.^[^ ^4^, ^13^, ^51^ ^]^ Moreover, although the oxygen content in the C/SiOC bilayer may further increase the irreversible capacity by trapping sodium ions during the initial sodiation/desodiation processes, as shown in Figure S8a (Supporting Information),^[^ ^31^, ^50^ ^]^ the initial CE of Sn@C/SiOC is higher than that of Sn/C. This result indicates that the C/SiOC bilayer ensures higher usage of the Sn active material, good charge/discharge reversibility, and facile charge transport by virtue of its superior electrical conductivity.^[^ ^33^ ^]^ In addition, the low initial CE would be further improved through advanced presodiation strategy and electrolyte optimization research.^[^ ^1^, ^4^ ^]^ The conducive effects of the bilayer can be verified in the subsequent second cycle. Compared to the initial cycle, a higher desodiation capacity of the Sn/C electrode is observed, which is a general phenomenon arising from the sluggish activation process of the Sn anode.^[^ ^52^ ^]^ Conversely, the Sn@C/SiOC electrode shows well‐overlapping voltage profiles in the subsequent cycle, implying that the conductive C/SiOC bilayer ensures enhanced charge/discharge reversibility with a faster activation process. And, the electrochemical evaluation results, as presented in Figure S7 (Supporting Information), do not indicate a significant difference even at higher loading levels (≈4 mg cm^−2^). This result is considered significant in terms of ensuring the practical applicability of the as‐prepared material. The detailed reaction mechanism of Sn with sodium is elucidated by the differential capacity (d*Q*/d*V*) curves (Figure 3b; and Figure S6b, Supporting Information) derived from the charge/discharge voltage profiles. The d*Q*/d*V* curves of both Sn‐based electrodes yielded four sodiation peaks at ≈0.35, 0.18, 0.08, and 0.01 V on discharge, corresponding to continuous Na*─*Sn alloying reactions (formation of NaSn_5_, NaSn, Na_9_Sn_4_, and Na_15_Sn_4_).^[^ ^6^, ^7^, ^14^, ^54^, ^55^ ^]^ Upon reverse charging, four desodiation peaks at ≈0.12, 0.16, 0.25, and 0.53 V were observed, indicating the typical dealloying characteristics of Na*─*Sn alloys.^[^ ^6^, ^7^, ^14^ ^]^ All cathodic/anodic reactions of the Sn/C electrode are slightly delayed compared to those of Sn@C/SiOC. This result is attributed to the sluggish electrochemical kinetics of Sn/C due to the absence of the conductive coating layer.

The cycling performance of the Sn‐based electrodes is measured at a high current density of 1 A g^−1^ for 400 cycles, as shown in Figure 3c. The Sn/C electrode exhibited unstable cycling behavior with a capacity retention of only 51.3% after 400 cycles because of the large volume changes during repeated cycling. The well‐known capacity degradation mechanism of the high‐capacity anode material Sn is as follows: Initially, during the first sodiation process, an SEI layer forms on the surface of Sn, followed by the formation of a Na_15_Sn_4_ alloy.^[^ ^6^, ^7^ ^]^ This process involves a volume change of ≈400% and during the reverse desodiation process, Na returns to the cathode, causing the anode to contract. Repeating these significant volume changes multiple times leads to the formation of cracks within the particles.^[^ ^8^ ^]^ The fragile SEI layer is continuously decomposed and regenerates on the newly exposed particle surface, increasing the irreversible capacity. This series of processes gradually reduces the reversible capacity of the anode and can ultimately lead to the failure of the electrode as the anode delaminates from the current collector.^[^ ^4^, ^11^, ^12^, ^13^, ^14^ ^]^ In contrast, the Sn@C/SiOC electrode retained a high reversible capacity of ≈90% of its initial value even after 400 cycles. In order to determine the optimal C/SiOC bilayer content, various composite materials were synthesized by changing the ratio of silicone oil and SnO_2_/PDA precursors (SnO_2_/PDA: silicone oil = 1.0 g: 2.0 g (low C/SiOC content), 1.0 g: 4.0 g (this work), and 1.0 g: 6.0 g (high C/SiOC content)). As shown in Figure S9 (Supporting Information), the thick coating layer (33.4 wt% of C/SiOC) resulted in excellent structural stability for the core material; however, the overall specific capacity decreased because of the low capacity of the coating material. Furthermore, the composite material with a thin coating layer (7.6 wt% of C/SiOC) may result in higher capacity but poor cycling stability because of the insufficient protective effects. Thus, it was believed that the composite material with the moderate C/SiOC content (13.1 wt% of C/SiOC) optimized in this work should be used to achieve the highest cell performance. Additionally, the CE rapidly exceeded 99% in the first few cycles and maintained almost 100% until 400 cycles, confirming the significantly improved performance. The enhanced cycling stability can be attributed to the presence of the robust C/SiOC bilayer and a void interspace inside the hybrid structure, which effectively accommodates the huge volume changes of the Sn yolk. As discussed above, during the long‐term cycling process, the well‐controlled yolk–shell structure ensures stable electrochemical reactions without structural deterioration. In addition, the C/SiOC bilayer has a surface‐based sodium‐ion storage characteristic without capacity fading even after 400 cycles (Figure S8b, Supporting Information). Interestingly, the capacity decreases slightly and increases within the first several cycles, which was be observed for certain heterostructured hybrid materials. The capacity climbing can be described by the gradual activation process of the hybrid materials. The agglomerated sites, which did not participate in the electrochemical reaction for the initial few cycles, gradually take off each other, which induces facile sodium‐ion insertion/extraction and offers more accessible sites for sodium ions, further increasing the specific capacity.^[^ ^6^ ^]^ This ensures a higher reversible capacity of the Sn‐based electrode. The ex situ XRD analyses of the Sn@C/SiOC electrode confirmed the disappearance of Sn peaks with reversible evolution of Na_15_Sn_4_ peaks at the fully sodiated states after 30 and 400 cycles (Figure 3d), demonstrating the maintenance of stable alloying reactions between sodium and Sn over remarkably extended cycling. Additionally, the Sn@C/SiOC electrode possesses a superior rate performance compared to the Sn/C (Figure 3e). The as‐prepared Sn@C/SiOC nanohybrid electrode delivered 521.3, 502.7, 493.4, 483.4, 481.3, 472.4, 442.4, and 419.6 mAh g^−1^ at current densities of 0.05, 0.1, 0.2, 0.5, 1.0, 2.0, 5.0, and 10 A g^−1^, respectively, whereas the corresponding specific capacities of the Sn/C electrode were 511.8, 502.8, 499.0, 461.2, 459.1, 267.4, 0.4, and 0.2 mAh g^−1^, respectively. At current densities lower than 1.0 A g^−1^, both Sn‐based electrodes show no drastic differences in reversible capacities. However, a significant capacity drop was observed for the Sn/C electrode as the current density surpassed 1.0 A g^−1^, and this electrode cannot function as a host material for SIBs at current densities of 5.0 and 10 A g^−1^. Conversely, even at a high current density of 10 A g^−1^, the Sn@C/SiOC electrode exhibited a remarkably higher capacity retention, over 80% of the reversible capacity at 0.05 A g^−1^. In addition, the Sn@C/SiOC electrode exhibited a better capacity recovery phenomenon at the low current density of 0.5 A g^−1^ following the rate performance test compared to the pure Sn/C electrode. The galvanostatic charge/discharge voltage profiles of the Sn/C and Sn@C/SiOC electrodes obtained at various current densities from 0.05 to 10 A g^−1^ were compared to understand the superior rate capability of the Sn@C/SiOC electrode (Figure S10, Supporting Information). Most alloy‐based anodes reveal drastic voltage hysteresis after sodiation/desodiation processes, particularly at high charge/discharge rates, due to their slow electrochemical kinetics caused by unstable SEI formation and sluggish alloying/dealloying reactions with sodium.^[^ ^1^ ^]^ Similarly, the Sn/C electrode demonstrated a large voltage hysteresis as the current density increased. Conversely, the Sn@C/SiOC anode exhibited outstanding sodiation/desodiation reversibility with smaller overpotentials even at a high current density of 10 A g^−1^.

The variation of the electrode surface morphology of the Sn‐based electrodes after prolonged cycling was investigated using postmortem FE‐SEM analysis by disassembling the coin cells. As shown in Figure 3f, the top‐view SEM image of the pure Sn/C electrode after 400 cycles shows the generation of massive cracks and structural disintegration due to particle agglomeration and pulverization through the repeated alloying/dealloying reactions with significant volume changes of active materials. Even after 400 cycles, the Sn@C/SiOC electrode retains a smooth, uniform surface without macro‐cracks or electrode degradation (Figure 3g), confirming the effectiveness of the yolk–shell nanohybrid structure on the mechanical stability. Moreover, the microstructural changes of the Sn‐based active materials were examined by ex situ TEM and corresponding EDS mapping images. After 400 cycles, the Sn@C/SiOC particles exhibited well‐maintained yolk–shell morphology without structural deterioration (insets of Figure 3g; and Figure S11, Supporting Information) compared to the Sn/C particles that are agglomerated with a nonuniform size distribution (inset of Figure 3f).

Electrochemical impedance spectroscopy (EIS) analysis was conducted to check the origin of the improved electrochemical behavior of the Sn@C/SiOC electrode. Figure 3h,i shows the comparison of the Nyquist plots collected at the fully desodiated state (0.8 V vs Na/Na^+^) for both Sn‐based electrodes before cycling and after 30 and 400 cycles. The fitted values of each resistance component, such as electrolyte resistance *R*_ele_, interfacial resistance *R*_int_, and charge transfer resistance *R*_ct_, using an equivalent circuit model are listed in Table S2 (Supporting Information). In the pristine state, both Sn‐based electrodes yielded similar *R*_ele_ values (< 2 Ω); whereas the *R*_ct_ value of Sn/C (536.4 Ω) was greater than that of Sn@C/SiOC (440.5 Ω), indicating a higher electrochemical barrier for sodium‐ion transport, which can be confirmed by comparing the initial discharge voltage curves of both electrodes, as shown in Figure 3a; and Figure S6a (Supporting Information). The initial *iR* drop of the Sn@C/SiOC electrode is lower than that of Sn/C, demonstrating that the mesoporous and conductive bilayered shell lowers the activation barrier and facilitates alloying reactions between Sn and sodium. After prolonged cycling, all resistance values of Sn@C/SiOC were consistent, even after 30 and 400 cycles (Figure 3i). However, the *R*_int_ and *R*_ct_ Sn/C electrode values significantly increased upon cycling (Figure 3h), which is mainly attributed to particle pulverization, the repetitive formation of an unstable SEI layer, and the absence of the conductive coating layer that hampers the fast transportation of sodium ions and electrons; this results in sluggish electrochemical kinetics and mechanical failure with severe capacity fading. Therefore, the EIS results indicate that the C/SiOC bilayer stabilizes the interface between the electrode and electrolyte and promotes electrochemical reaction kinetics, contributing to enhanced cycling and rate capabilities.

#### 2.3.2 Study on Sodium‐Ion Diffusion Kinetics and Reaction Mechanism

**Figure 4:**
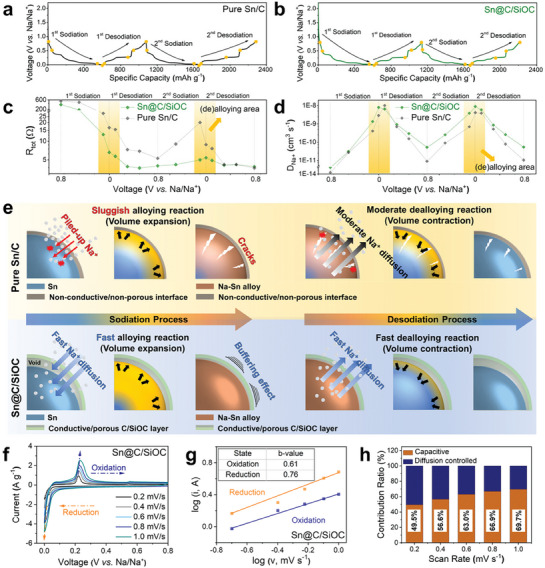
In situ EIS analysis of the Sn‐based electrodes at various state‐of‐charge (SOC) conditions. Charge–discharge voltage profiles for the initial two cycles of the a) pure Sn/C and b) Sn@C/SiOC electrodes were measured at 0.05 A g^−1^ (≈0.07 C‐rate) with voltage points where EIS was taken. Variation of the (c) total resistance (*R*_tot_) and d) sodium‐ion diffusion coefficient (*D*_Na⁺_) values of the pure Sn/C and Sn@C/SiOC electrodes measured at voltage points indicated in a) and b). e) Schematic illustration of the sodiation/desodiation mechanisms of the pure Sn/C and Sn@C/SiOC electrodes. f) CV curves at different sweep rates (0.2–1.0 mV s^−1^) and g) log *i* versus log *v* plots of the Sn@C/SiOC electrode (inset table: estimated b‐values). h) Comparison of the relative contribution ratio of capacitive‐ and diffusion‐controlled kinetics at various sweep rates (0.2–1.0 mV s^−1^).

In situ EIS analysis at various state‐of‐charge (SOC) conditions were conducted to further understand the electrochemical kinetics of the Sn‐based electrodes. **Figure** **4**a,b represents the charge–discharge voltage profiles of both Sn‐based electrodes at 0.05 A g^−1^ for the initial two cycles, with several potential points where Nyquist plots are obtained. Based on the Nyquist plots obtained at various SOC conditions (Figure S12, Supporting Information), the measured resistance values and calculated diffusion coefficient *D*_Na⁺_ are summarized in Tables S3 and S4 (Supporting Information). The value of the total resistance (*R*_tot_ = *R*_ele_ + *R*_int_ + *R*_ct_) was estimated, and the *D*_Na⁺_ values were determined using Equation (1) to compare the changes in the resistance and *D*_Na⁺_

(1) $$D_{Na^{+}}=R^{2}T^{2}/2A^{2}n^{4}F^{4}C^{2}{\sigma_{w}}^{2}$$

where *R* and *T* are the gas constant (8.314 J mol^−1^ K^−1^) and absolute temperature (298.15 K), respectively, and *A*, *n*, *F*, and *C* refer to the electrode surface area, a number of electrons involved in the electrochemical reaction, Faraday constant (96,485 C mol^−1^), and the sodium‐ion concentration, respectively. The Warburg impedance coefficient (*σ*_w_) is determined by the slope of the linear fitting of real impedance (Z′) versus ω^−0.5^ plots in the Warburg term at low‐frequency regions.

The changes in the *R*_tot_ and *D*_Na⁺_ values of the Sn/C and Sn@C/SiOC electrodes obtained from several SOCs during the initial two cycles are presented in Figure 4c,d, respectively. *R*_tot_ values of the Sn@C/SiOC electrode were lower compared to those of Sn/C at all SOC conditions, whereas both Sn‐based electrodes exhibited similar variation trends for each parameter. Additionally, the Sn@C/SiOC electrode has higher overall *D*_Na⁺_ values than the pure Sn/C electrode under identical SOCs. This indicates that the sodium‐ion transportation capability of Sn@C/SiOC is superior to that of the Sn/C electrode during battery operations. Moreover, as evident from Figure 4c,d, a significant gap between two Sn‐based electrodes is observed for both *R*_tot_ and *D*_Na⁺_ values during the alloying/dealloying reactions (yellow box area). Alloy‐based anode materials, such as Si and Sn,^[^ ^56^, ^57^ ^]^ have generally exhibited asymmetric alloying/dealloying behavior that originated mainly from a sluggish alloying step, which eventually causes poor rate performance. Compared to the desodiation process, the Sn/C electrode revealed higher *R*_tot_ and lower *D*_Na⁺_ values in sodiation, confirming that the Na*─*Sn alloying process is the rate‐determining step for the sodiation/desodiation of the Sn anode. The Sn@C/SiOC electrode demonstrated substantially lower *R*_tot_ and higher *D*_Na⁺_ values compared to Sn/C, particularly in the first two cycles. Therefore, the realization of the hierarchical heterostructured nanohybrid composed of Sn yolk and C/SiOC shell provides a great boost for high‐rate sodium‐ion storage performance.

Figure 4e shows the different reaction mechanisms between the Sn/C and Sn@C/SiOC electrodes. For the Sn/C electrode, the sodiation gradually proceeds because of the slow diffusion kinetics without the porous ionic transport channels at the particle surface, whereas a relatively faster sodium‐ion extraction can be achieved during desodiation. However, huge volume expansion/contraction of Sn causes undesirable surface cracking, pulverization of particles upon alloying/dealloying, and the generation of large Sn agglomerates after prolonged cycles,^[^ ^9^, ^12^, ^28^ ^]^ which may increase the effective diffusion distance and further interfere with redox reactions between sodium and Sn. For the Sn@C/SiOC electrode, the mesoporous and conductive C/SiOC shell offers a facile ionic transfer network at the particle surface, which facilitates the movement of sodium ions and ensures fast diffusion kinetics upon cycling. Furthermore, the mechanically and chemically stable C/SiOC shell relieves the internal mechanical stress arising from the large Sn yolk volume change and prevents the agglomeration of Sn particles and unfavorable side reactions between Sn and organic electrolyte, thereby enhancing the structural stability and electrochemical diffusion kinetics.

The electrochemical kinetics of the Sn@C/SiOC electrode was investigated via CV tests at various scan rates (0.2–1.0 mV s^−1^) to elucidate the reaction mechanism, as shown in Figure 4f; and Figure S13a (Supporting Information). The charge storage mechanism of Sn consists of two kinetic factors, such as diffusion‐ and capacitive‐controlled processes,^[^ ^58^, ^59^ ^]^ and their relative contributions can be quantified using Equation (2)

(2) logi=loga+blogv

where *v* is the sweep rate, *i* is the measured peak current, and *a* and *b* are the empirical parameters. The *b*‐values for each sodiation and desodiation process can be determined from the slope of the linear relationship of the log *i* versus log *v* plot, as shown in Figure 4g; and Figure S13b (Supporting Information). Generally, the electrochemical reaction is dominated by surface capacitive behavior when the *b*‐value is closer to 0.5, whereas the charge storage process is predominantly influenced by the diffusion‐controlled faradaic behavior when the *b*‐value is closer to 1.0.^[^ ^57^, ^59^ ^]^ The calculated *b*‐values of the Sn@C/SiOC electrode are 0.61 and 0.76 for the oxidation and reduction steps, respectively, indicating combined diffusion‐controlled and capacitive sodiation/desodiation behaviors (Figure 4g). Conversely, the Sn/C electrode exhibits *b*‐values of 0.5 and 0.61 for the oxidation and reduction reactions, respectively, revealing that most sodium ions can be stored through the diffusion‐controlled process (Figure S13b, Supporting Information). In addition, the relative contribution ratios of capacitive‐ and diffusion‐controlled kinetics at various sweep rates are calculated and summarized in Figure 4h using Equation (3)^[^ ^33^ ^]^

(3) $$i=k_{1}v+k_{2}v^{1/2}$$

the values of *k*_1_ *v* (capacitive‐controlled) and *k*_2_ *v* ^1/^ *^2^* (diffusion‐controlled) were determined by the measured current (*i*) and sweep rate (*v*) using Equation (3). When the sweep rate was increased from 0.2 to 1.0 mV s^−1^, the capacitive‐controlled contribution ratio increased from 49.5% to 69.7%. This result may be attributed to the mesoporous structure of the C/SiOC bilayer, which provides additional sodium‐ion adsorption/desorption sites via capacitive‐controlled kinetics, boosting sodium‐ion storage characteristics even under fast charge/discharge conditions.^[^ ^33^ ^]^

**Figure 5:**
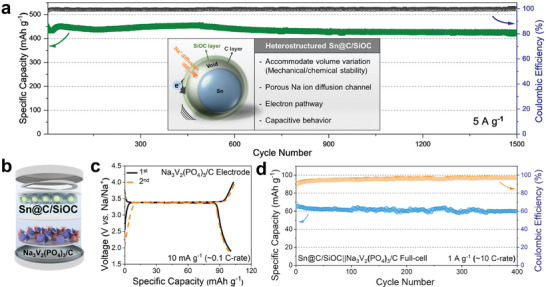
a) Long‐term cycling performance of the Sn@C/SiOC electrode at a current density of 5 A g^−1^ and schematic of the combined effects of the multifunctional C/SiOC bilayer and yolk‐shell nanohybrid structure (inset). b) Coin‐type full‐cell configuration consisting of Sn@C/SiOC as anode and Na_3_V_2_(PO_4_)_3_/C as cathode. c) Galvanostatic charge–discharge voltage profiles for the 1st and 2nd cycles of the Na_3_V_2_(PO_4_)_3_/C as cathode measured at 0.01 A g^−1^(≈0.1 C‐rate, 1 C‐rate for Na_3_V_2_(PO_4_)_3_: 117 mAh g^−1^). d) Cycling performance of the full‐cell at a current density of 1 A g^−1^ (≈10 C‐rate).

Furthermore, as shown in **Figure** **5**a, the nanohybrid electrode demonstrates ultrastable cycling performance up to 1500 cycles even at a high current density of 5 A g^−1^ (≈7 C‐rate) with a remaining capacity of 421.1 mAh g^−1^ (retention: 91.9%), which is outstanding sodium‐ion storage performances compared to the previous studies related to the Sn‐based anodes for SIBs (Table S5, Supporting Information).^[^ ^60^, ^61^, ^62^, ^63^, ^64^, ^65^ ^]^ With the above physicochemical and electrochemical characterization, the reasons for obtaining excellent sodium‐ion storage performance from the Sn@C/SiOC nanohybrid are summarized, as shown in the inset of Figure 5a. First, through the sodiation and desodiation processes, the abundant void interspaces between the Sn yolk and C/SiOC bilayer shell inside the nanohybrid particles could effectively accommodate severe volume changes of Sn, leading to structural reinforcement and enhanced mechanical stability of the nanohybrid electrode. Second, the mesoporous and conductive C/SiOC shell facilitates the electrolyte's penetration into the inner Sn yolk and promotes the transport of sodium ions and electrons, ensuring fast sodium‐ion diffusion kinetics and rapid redox reactions. Finally, this mesoporous C/SiOC structure provides additional sites for storing sodium ions through a capacitive reaction, enabling the fabrication of high‐energy density anodes for SIBs.

#### 2.3.3 Full‐Cell Application

Finally, to confirm the potential application of the Sn@C/SiOC nanohybrid anode, a full‐cell SIB was fabricated using Na_3_V_2_(PO_4_)_3_/C as the cathode (Figure 5b). Prior to fabrication, the Sn@C/SiOC electrode was precycled and presodiated, and the Na_3_V_2_(PO_4_)_3_/C electrode also precycled in a half‐cell condition. The Na_3_V_2_(PO_4_)_3_/C cathode was prepared via the sol–gel method and demonstrates charge/discharge capacity of 103.0/100.5 mAh g^−1^ with 97.6% initial CE at a current density of 10 mA g^−1^ (Figure 5c).^[^ ^34^ ^]^ The Sn@C/SiOC || Na_3_V_2_(PO_4_)_3_/C full‐cell shows a reversible capacity of ≈60 mAh g^−1^ at a current density of 1 A g^−1^ with superior cycling stability over 400 cycles. Thus, the superior electrochemical performance of the Sn@C/SiOC nanohybrid in full‐cell shed light on successful implementation of advanced SIB technology.

## 3 Conclusions

We synthesized the hierarchically designed yolk–shell Sn@C/SiOC via a facile pyrolysis technique for the first time to overcome the sluggish sodiation kinetics and massive volume changes of the Sn anode. The combined microstructural and morphological characterization results revealed that the Sn‐based nanohybrids were composed of nanoscale Sn yolk particles with an interfacial SnO*_x_* nanolayer, encapsulated within a multifunctional C/SiOC bilayer shell. The unique, well‐designed yolk–shell nanohybrid architecture provided abundant void interspaces between the nanosized Sn yolk and C/SiOC shell, mitigating large volume variations and enhancing the mechanical integrity and flexibility of the anode. Furthermore, the mesoporous C/SiOC bilayer further alleviated the massive volume change of Sn and enabled rapid sodium‐ion diffusion, particularly during the sodiation process. Therefore, the Sn@C/SiOC electrodes demonstrated excellent sodium storage performance in terms of high reversible capacity (521 mAh g^−1^), superior rate capability (up to 10 A g^−1^), and ultrastable cycling performance (5 A g^−1^ with a capacity retention of 91.1% even after 1500 cycles). The Sn@C/SiOC || Na_3_V_2_(PO_4_)_3_/C full‐cell with highly stable sodium‐ion storage performance over 400 cycles further demonstrates the practical high applicability of the high‐capacity anode. The simple and facile synthetic procedure and hierarchical design approach based on the proposed yolk–shell heterostructure offer valuable insights into the development of various electrode materials for secondary batteries and energy storage devices.

## Conflict of Interest

The authors declare no conflict of interest.

## Supporting information



Supporting Information

## Data Availability

The data that support the findings of this study are available from the corresponding author upon reasonable request.
