# Supplementary material for: Boosting the Sodiation Kinetics of Sn Anode Using a Yolk–Shell Nanohybrid Structure for High‐Rate and Ultrastable Sodium‐Ion Batteries
Source: Adv Sci (Weinh). 2024 Oct 31;11(48):2408450. doi: 10.1002/advs.202408450 (PMC11672318; doi:10.1002/advs.202408450)
Supplement: Supplementary file 1 — Supporting Information [file ADVS-11-2408450-s001.docx]

Supporting Information

Boosting the Sodiation Kinetics of Sn Anode using a Yolk-Shell Nanohybrid Structure for High-Rate and Ultrastable Sodium-Ion Batteries

Hyojun Lim, Seungho Yu, Wonyoung Chang, Kyung Yoon Chung, Wonchang Choi*, and Sang-Ok Kim*

**Supporting Information**

Methods and Materials can be found here. Fourier transform-infrared spectra of the h-SnO_2_ and h-SnO_2_@PDA precursors; FE-SEM images of h-SnO_2_ and low-magnification bright-field TEM images of the h-SnO_2_ and h-SnO_2_@PDA precursors; low-magnification bright-field TEM image of the Sn/C hybrid without SiOC coating layer; Comparison of powder XRD patterns of the h-SnO_2_ and h-SnO_2_@PDA precursors; nitrogen adsorption–desorption isotherm SiOC; galvanostatic charge-discharge voltage profiles for the 1^st^ and 2^nd^ cycles of the pure Sn/C electrode measured at a current density of 0.05 A g^-1^ and corresponding dQ/dV curves obtained from the voltage profiles; galvanostatic charge-discharge voltage profiles for the initial cycle of the various Sn@C/SiOC electrodes with different loading levels measured at 0.05 A g^-1^; charge/discharge voltage profiles during the initial two cycles of SiOC between 0.001 V and 0.8 V *vs.* Na/Na^+^ at 50 mA g^-1^; the cycle performance of the SiOC anode material at 1 A g^−1^; TGA curves of various Sn-based materials and SiOC sample; TEM-EDS mapping image of the Sn@C/SiOC samples with the low SiOC content and high SiOC content; comparison of the cycling performance of the various Sn@C/SiOC samples; voltage profiles of pure Sn/C and Sn@C/SiOC electrodes at various current densities from 0.05 to 10 A g^-1^; TEM-EDS mapping image of the Sn@C/SiOC nanohybrid particles after 400 cycles; *in situ* EIS analysis of Sn-based electrodes at various state-of-charge (SOC) conditions; CV curves at different sweep rates and log *i* vs. log *v* plots of the pure Sn/C electrode; summary of fitted resistance values of the pure Sn/C and Sn@C/SiOC electrodes upon cycling measured using a simplified equivalent circuit; impedance parameters and diffusion coefficient values of the pure Sn/C and Sn@C/SiOC electrodes during the 1^st^ and 2^nd^ (de)sodiation processes at various voltages; Summary of sodium storage performance of various Sn-based anodes for SIBs.

**Material synthesis**

*Synthesis of the h-SnO_2_ precursor*: First, potassium stannate trihydrate (K_2_SnO_3_·3H_2_O, 0.768 g, 99.9%, Sigma-Aldrich) and urea (0.9 g, 98%, Sigma-Aldrich) were dissolved in 160 mL of deionized water/absolute ethanol (5:3, v/v) mixture solution under vigorous stirring for 30 min. Subsequently, the solution was transferred into a 200-mL Teflon-lined stainless autoclave and then maintained at 190 °C for 10 h. Finally, the h-SnO_2_ precursor was thoroughly washed, filtered several times, and dried at 60 °C for 12 h.

*Synthesis of the h-SnO_2_@PDA precursor*: The as-prepared h-SnO_2_ powders (0.5 g) were dispersed in 10 mM of tris-buffer aqueous solution (200 mL) by ultrasonication treatment for 30 min. Subsequently, an appropriate amount of dopamine hydrochloride (0.5 g, 98%, Sigma-Aldrich) was added to the aqueous dispersion under continuous stirring for 2 h. In this step, dopamine hydrochloride was self-polymerized in alkaline solutions (pH > 7.5), and the surface of the h-SnO_2_ particle was covered by *in situ* formed PDA owing to its strong adhesion properties. Thereafter, the h-SnO_2_@PDA precursor was collected through filtration and dried at 60 °C for 12 h.

*Synthesis of the Sn@C/SiOC nanohybrid*: The as-prepared h-SnO_2_@PDA precursor (1.0 g) was dispersed in a mixture precursor (4.0 g, silicone oil : divinylbenzene = 10 : 1, v/v) by ultrasonication and further stirred vigorously to achieve a homogeneous suspension. Subsequently, the two-step pyrolysis of silicone oil suspension was conducted in a tube furnace at 500 °C for 4 h and then at 900 °C for 1 h under an inert atmosphere (i.e., argon). Consequently, the final Sn@C/SiOC nanohybrids were ground using a Mini-mill grinder (Pulverisette 23, Fritsch, Germany) to obtain fine powders.

**Material characterization**

Field emission-scanning electron microscopy (FE-SEM) was performed using a Teneo Volume Scope (FEI, USA). High-resolution transmission electron microscopy (HR-TEM) equipped with energy dispersive X-ray spectroscopy (EDS) was conducted with a Talos F200X (FEI, USA) operated at an accelerating voltage of 200 kV. X-ray photoelectron spectroscopy (XPS, PHI5000 VersaProbe, ULVAC-PHI, Japan) was performed to confirm the chemical states of each element. XPS depth profile measurement was performed with Ar^+^ sputtering (40 nm min^-1^, 2 kV). X-ray powder diffraction (XRD) was conducted with a MiniFlex (Rigaku, Japan) using a Cu K*α* radiation source (*λ* = 1.5417 Å) in the *2θ* range from 10° to 90° with a scan rate of 4° min^−1^. Raman spectra were collected using an inVia Raman microspectrometer (Renishaw Inc., UK) with a laser beam of 523 nm. To calculate the Sn content in composite materials, thermogravimetric analysis (TGA) was conducted using SDT-Q600 equipment (TA Corp., USA). The Brunauer-Emmett-Teller (BET) surface area and pore size distribution were acquired by nitrogen adsorption-desorption using ASAP-2010 (Micromeritics, USA). An iS10 equipment (Thermo Fisher Scientific, USA) was used to obtain the Fourier transform-infrared (FT-IR) spectra.

**Electrochemical measurements**

The sodium-ion storage performance of the Sn-based electrodes was investigated by various galvanostatic or potentiostatic electrochemical tests within the voltage range of 0.001–0.8 V (*vs.* Na/Na^+^). A pure Sn/C composite was prepared by mechanical ball milling using pure Sn powder (87 wt%) with nanosized carbon (Super P, 13 wt%) instead of C/SiOC, and its electrochemical behavior was evaluated under the same conditions to confirm the effects of the hierarchically designed heterostructure of the Sn@C/SiOC nanohybrid. Coin-type cells (CR2032, Wellcos, Korea) were assembled in an Ar-filled glove box using Sn-based materials as active materials. The working electrodes were fabricated by casting slurries composed of 80 wt% active material, 10 wt% Super P (conductive agent), and 10 wt% of poly(acrylic acid) dissolved in deionized water (binder) onto the copper foil (current collector). The typical loading mass of electrodes was adjusted to 1.5–2.0 mg cm^−2^. Sodium metal and glass fiber (Whatman) were used as a counter or reference electrode and a separator, respectively. The organic electrolyte was 1 M NaPF_6_ dissolved in 1,2-Dimethoxyethane (DME, Sigma-Aldrich). Galvanostatic charge/discharge tests and cyclic voltammetry (CV) measurements were performed in the voltage range of 0.001–0.8 V (vs. Na/Na^+^) at 25 °C. In addition, the specific capacity was calculated based on the total weight of the active material in the electrode. Electrochemical impedance spectroscopy (EIS) spectra were collected using a VSP-300 Potentiostat (Bio-logic, France) in the frequency range from 10 mHz to 1 MHz with an AC amplitude of 5 mV. As for the full-cell, the Na_3_V_2_(PO_4_)_3_/C cathode electrodes were fabricated by casting slurries composed of 80 wt% active materials, 10 wt% Super P (conductive agent), and 10 wt% of polyvinylidene fluoride (binder) onto the aluminum foil (current collector). Prior to fabrication, the Sn@C/SiOC anode was pre-cycled and pre-sodiated, and Na_3_V_2_(PO_4_)_3_/C cathode was also pre-cycled in a half-cell condition. Galvanostatic charge/discharge measurements of cathode electrodes and full-cells were carried out in the voltage ranges of 1.9–4.0 V and 2.0-3.7 V (vs. Na/Na^+^) at 25 °C, respectively. The specific capacity of the full-cell was calculated based on the loading mass of the cathode active material. (1 C-rate for Na_3_V_2_(PO_4_)_3_: 117 mAh g^-1^)


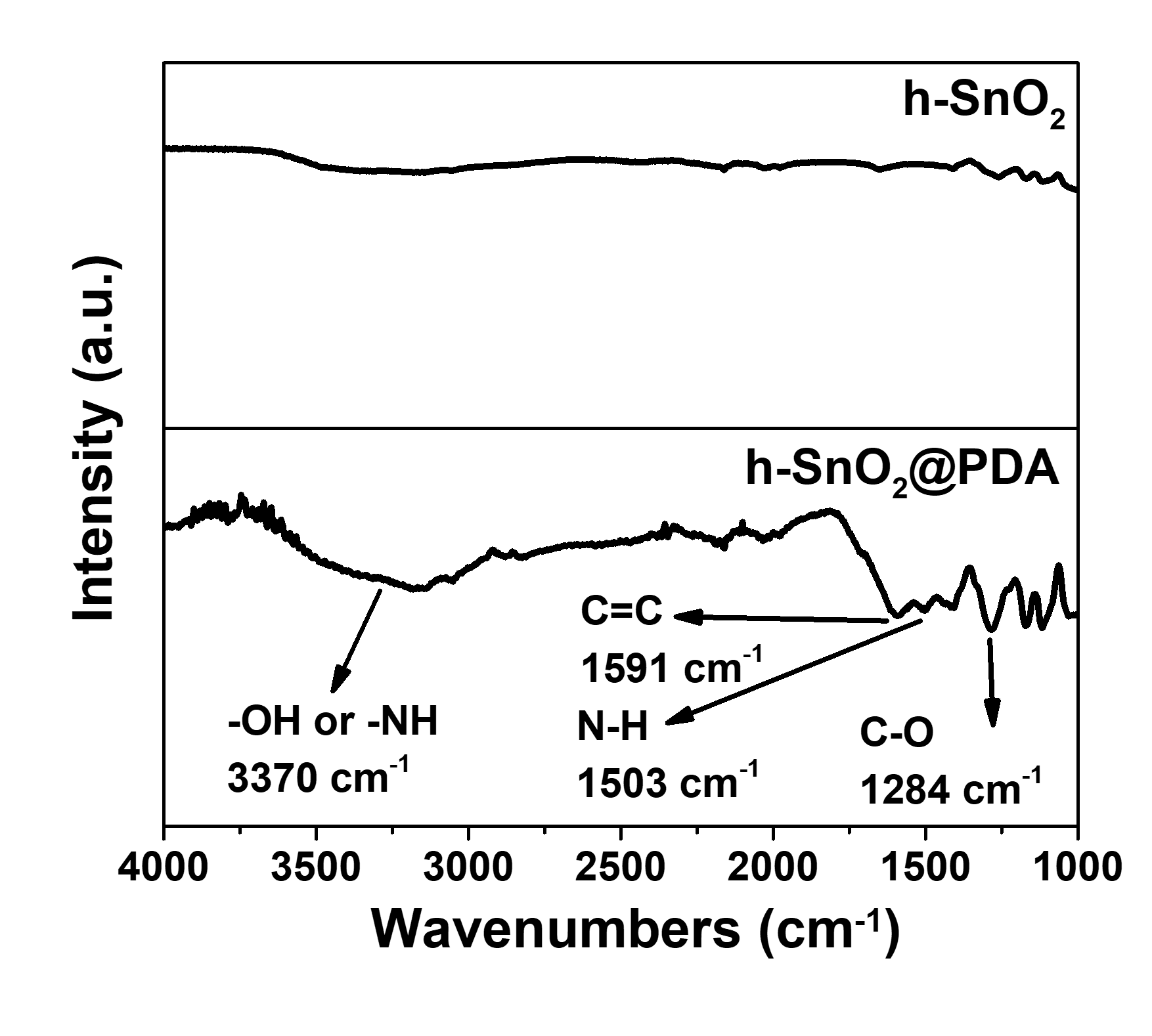


**Figure S1.** Fourier transform-infrared spectra of the h-SnO_2_ and h-SnO_2_@PDA precursors.


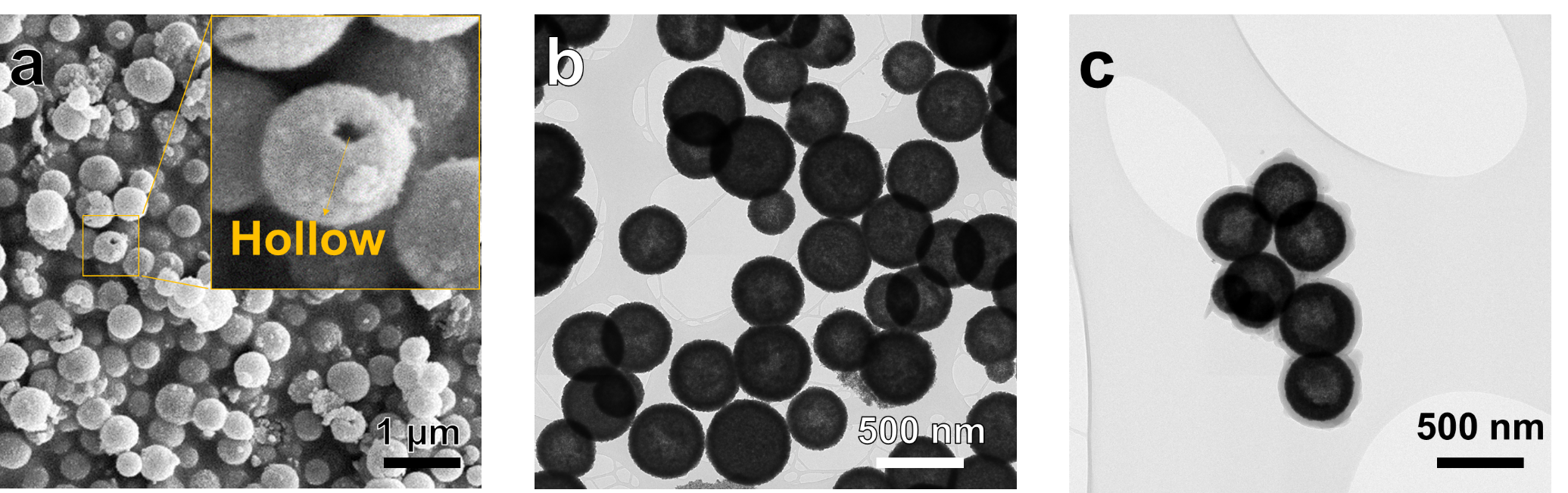


**Figure S2.** (a) FE-SEM image ands of h-SnO_2_. Low-magnification bright-field TEM images of the (b) h-SnO_2_ and (c) h-SnO_2_@PDA precursors.


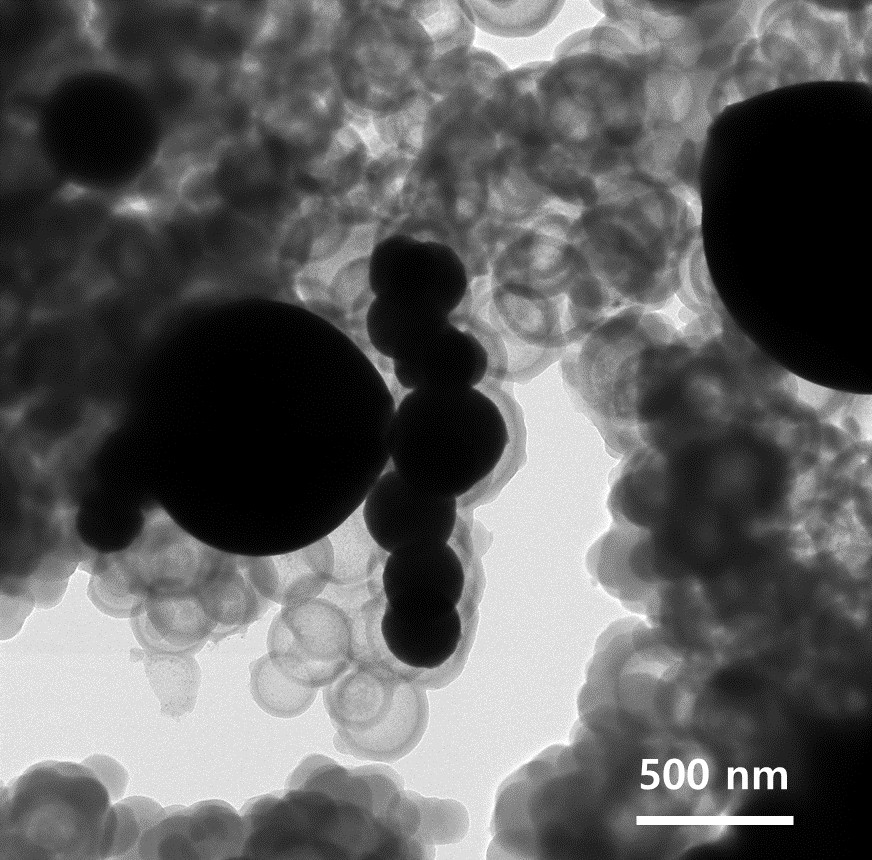


**Figure S3.** Low-magnification bright-field TEM image of the Sn/C hybrid without SiOC coating layer.


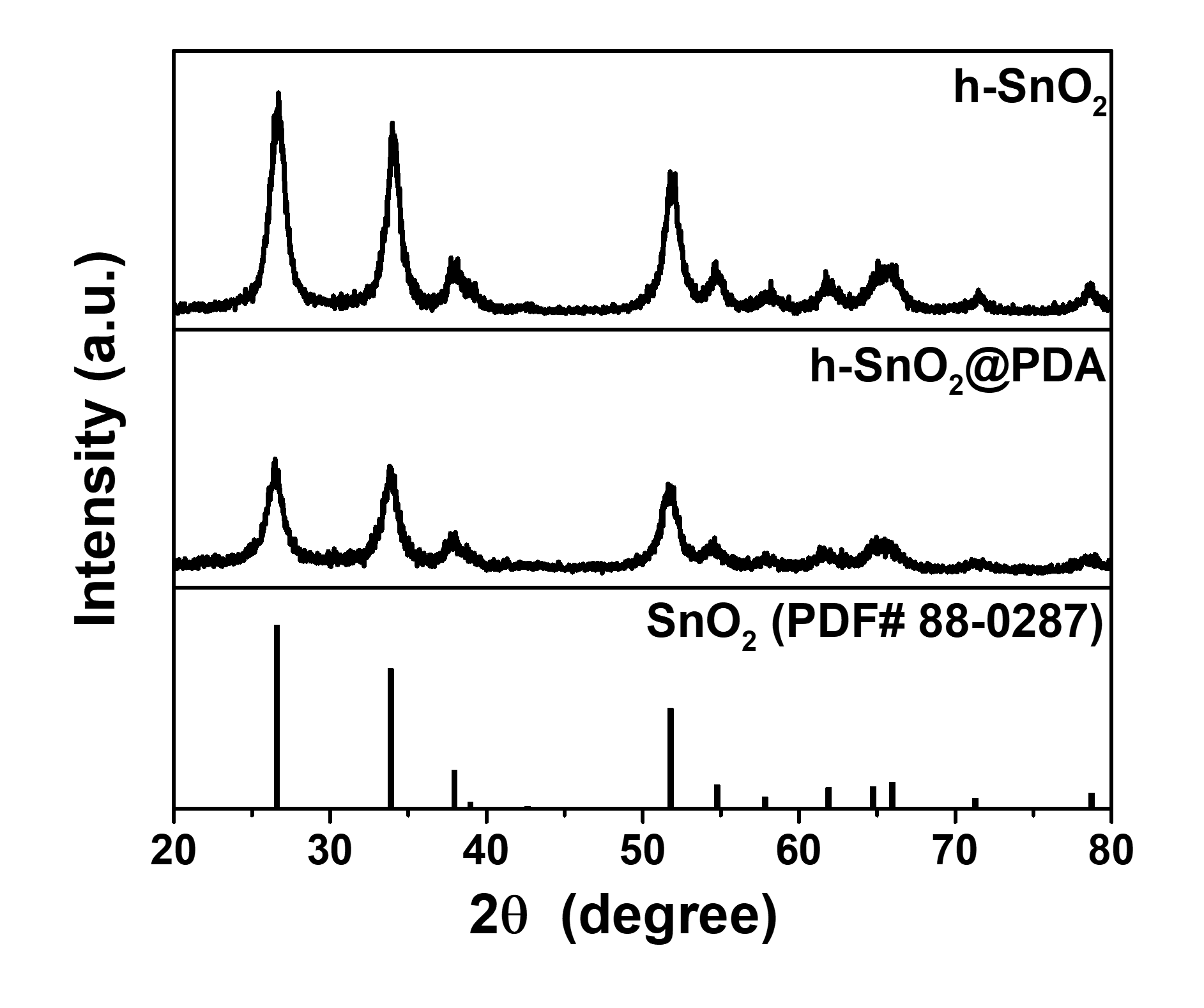


**Figure S4.** Comparison of powder XRD patterns of the h-SnO_2_ and h-SnO_2_@PDA precursors.

**
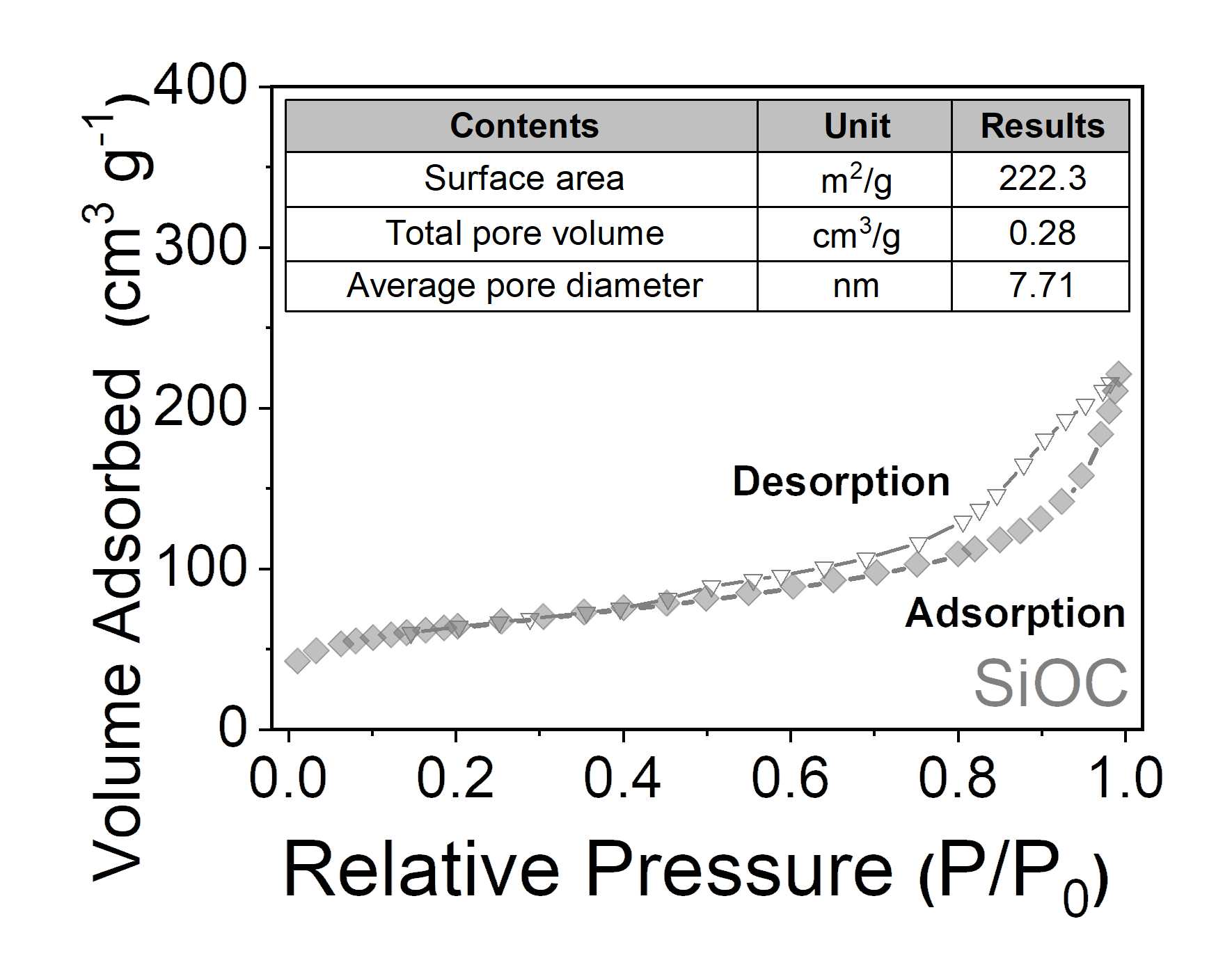
**

**Figure S5.** Nitrogen adsorption-desorption isotherm and surface information (inset) of SiOC.


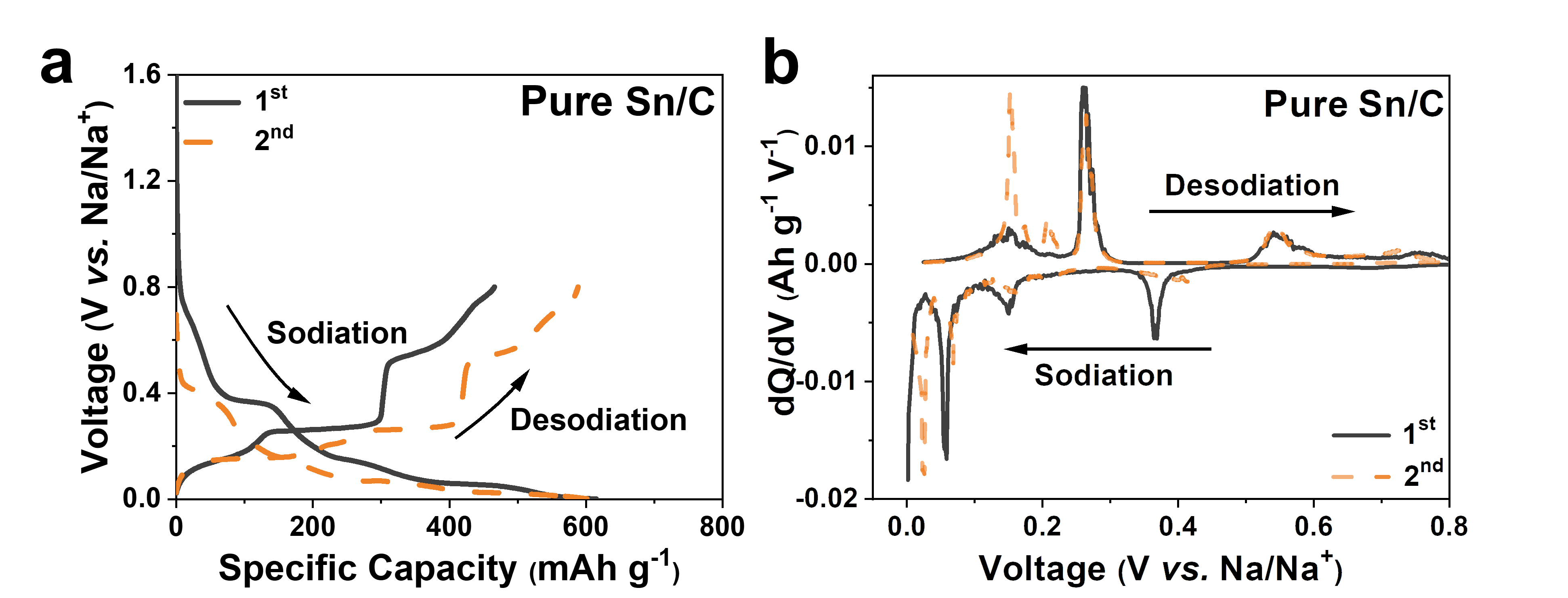


**Figure S6.** (a) Galvanostatic charge-discharge voltage profiles for the 1^st^ and 2^nd^ cycles of the pure Sn/C electrode measured at a current density of 0.05 A g^-1^ and (b) corresponding dQ/dV curves obtained from the voltage profiles in (a).


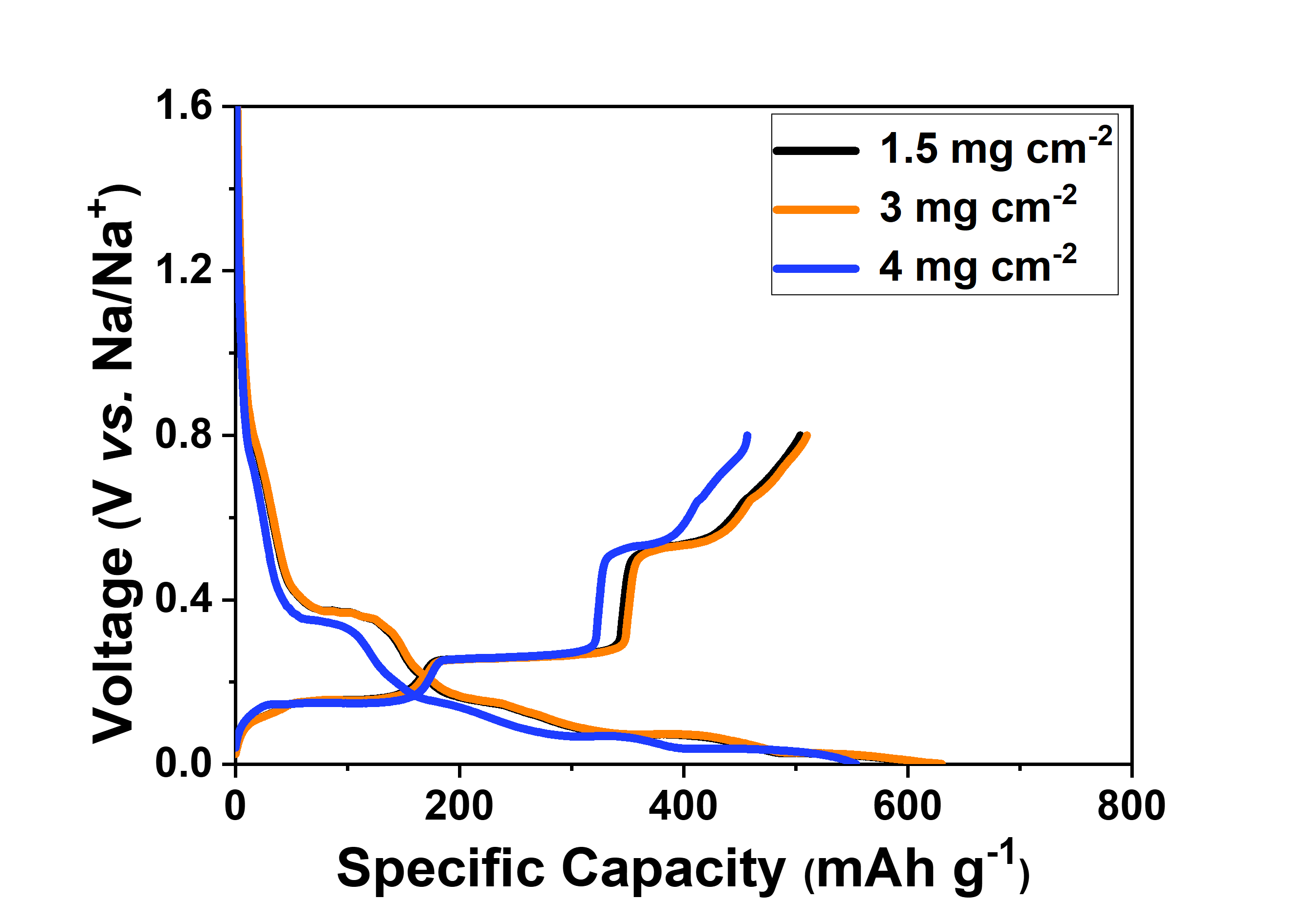


**Figure S7.** Galvanostatic charge-discharge voltage profiles for the initial cycle of the various Sn@C/SiOC electrodes with different loading levels measured at 0.05 A g^-1^.


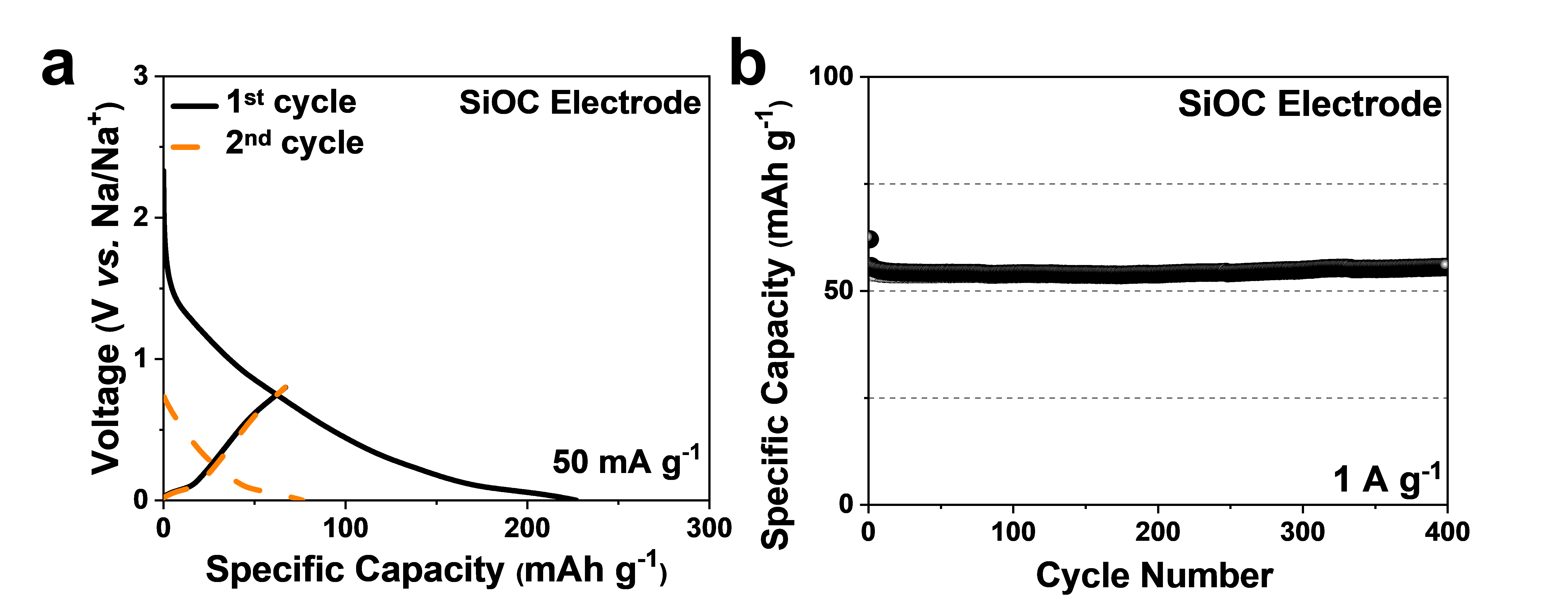


**Figure S8.** (a) Charge/discharge voltage profiles during the initial two cycles of SiOC between 0.001 V and 0.8 V *vs.* Na/Na^+^ at 50 mA g^-1^. (b) The cycle performance of the SiOC anode material at 1 A g^−1^.


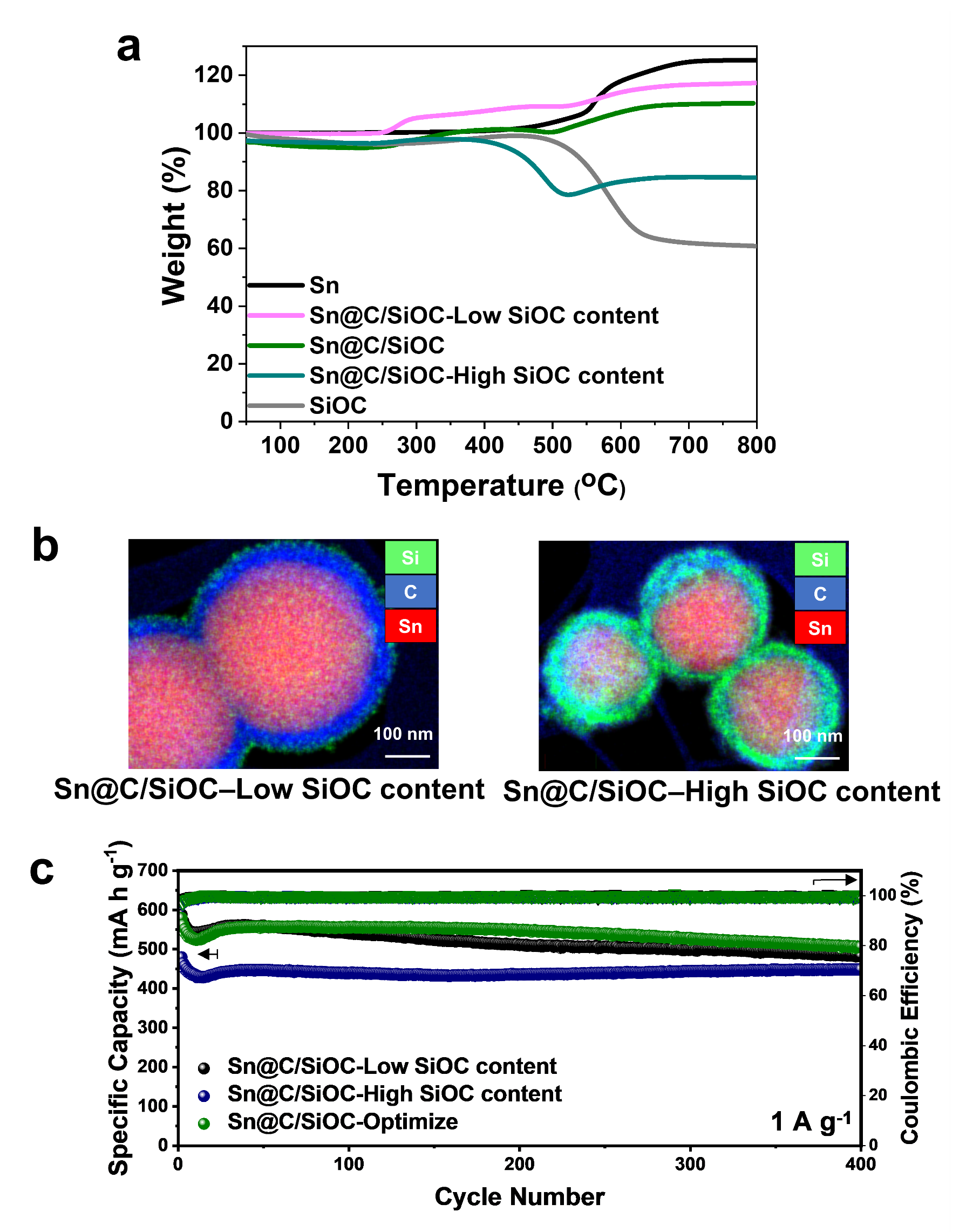


**Figure S9.** (a) TGA curves of various Sn-based materials and SiOC sample. (b) TEM-EDS mapping image of the Sn@C/SiOC samples with the low SiOC content and high SiOC content. (c) Comparison of the cycling performance of the various Sn@C/SiOC samples.

***
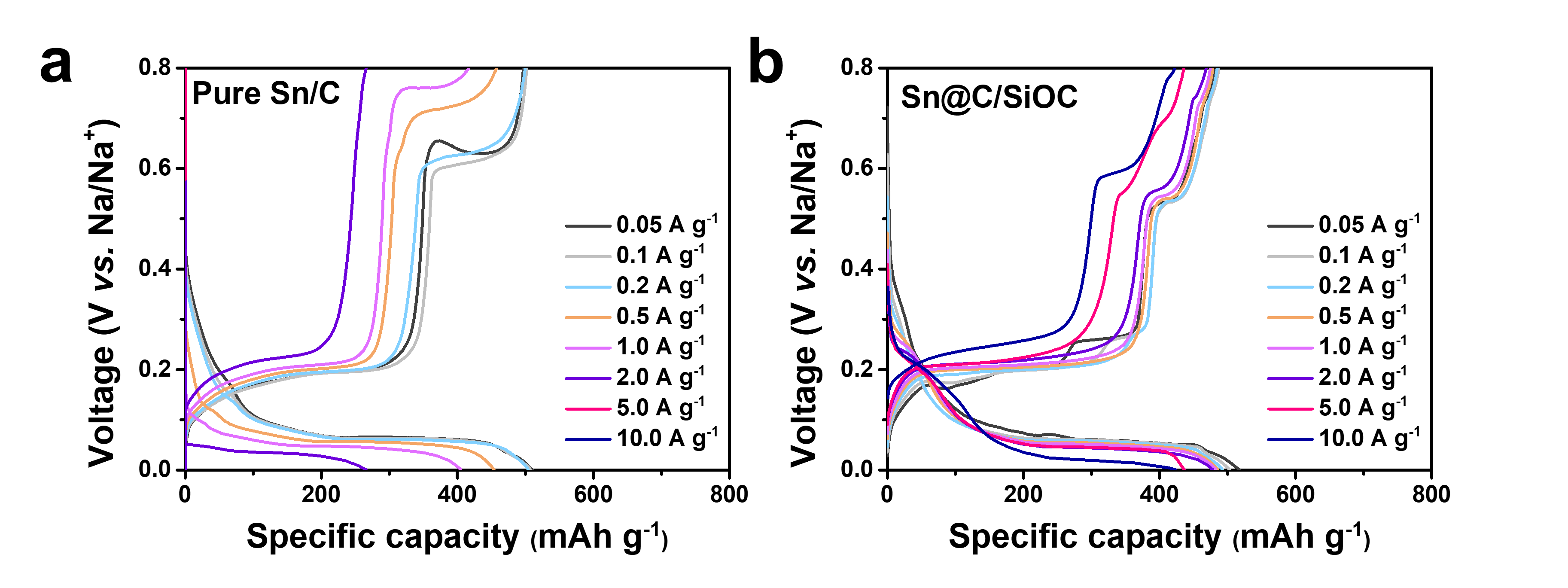
***

**Figure S10.** Voltage profiles of (a) pure Sn/C and (b) Sn@C/SiOC electrodes at various current densities from 0.05 to 10 A g^-1^.


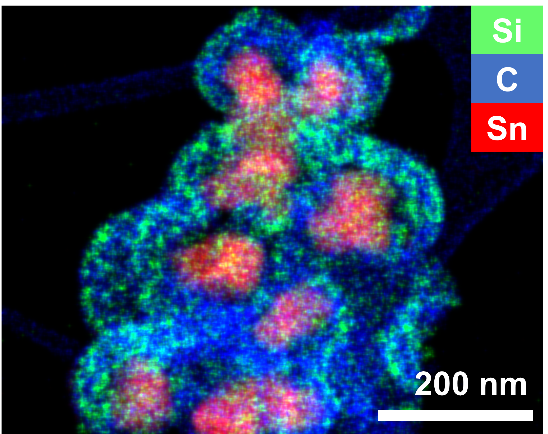


**Figure S11.** TEM-EDS mapping image of the Sn@C/SiOC nanohybrid particles after 400 cycles.

**
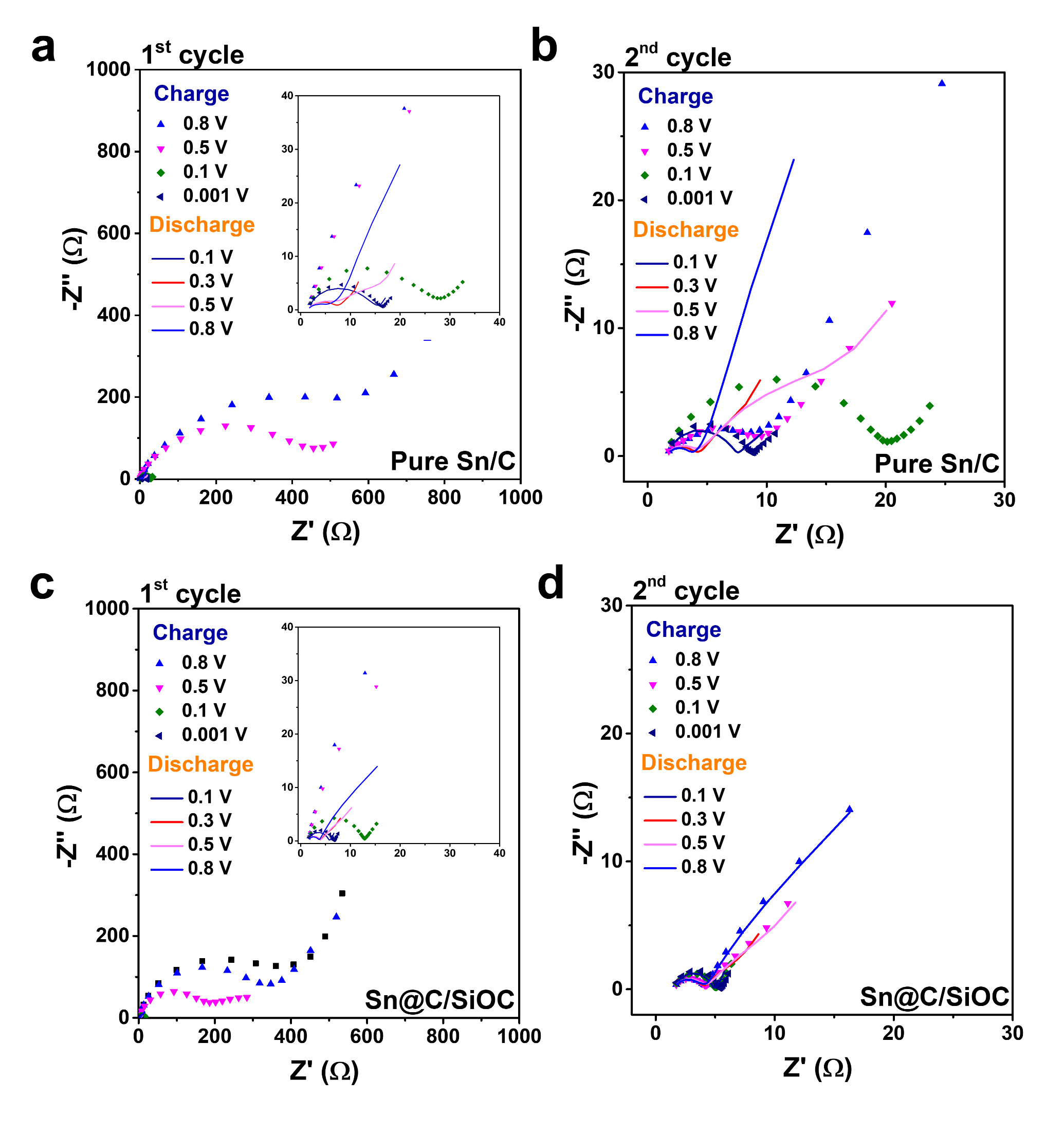
**

**Figure S12.** *In situ* EIS analysis of Sn-based electrodes at various state-of-charge (SOC) conditions: (a, b) First, second cycles of the pure Sn/C electrode and (c, d) first, second cycles of the Sn@C/SiOC nanohybrid electrode.


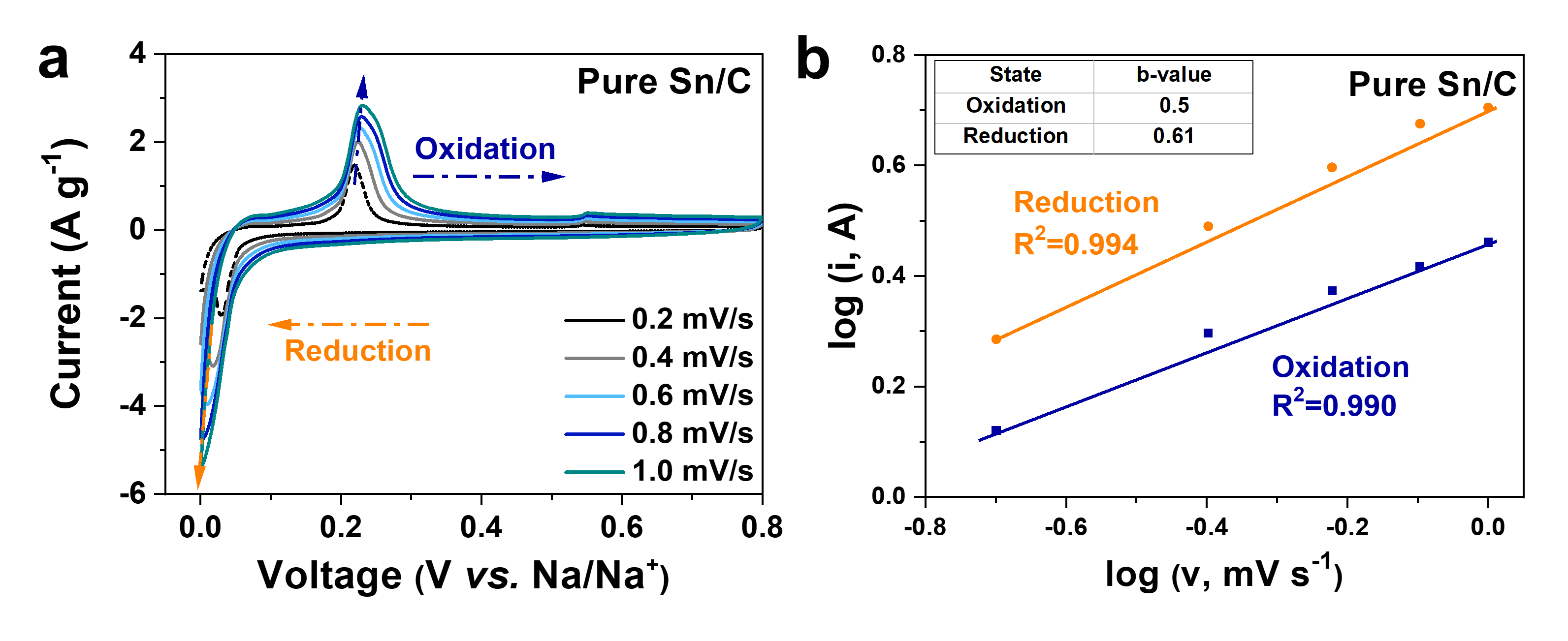


**Figure S13.** (a) CV curves at different sweep rates (0.2 – 1.0 mV s^-1^) and (b) log *i* vs. log *v* plots of the pure Sn/C electrode (inset table: estimated b-values).

**Table S1.** Electrochemical performance of Sn-based electrodes in SIBs.

| Sample | 1^st^ Charge capacity  (mAh g^-1^) | 1^st^ Discharge  capacity  (mAh g^-1^) | 1^st^ Coulombic efficiency (%) | Capacity retention (%) @ 400^th^ cycle | Capacity retention (%)  @ 10 A g^-1^ |
| --- | --- | --- | --- | --- | --- |
| Pure Sn/C | 615.0 | 466.1 | 75.8 | 51.3 | 0.2 |
| Sn@C/SiOC | 624.6 | 504.0 | 80.7 | 89.7 | 80.6 |

**Table S2.** Fitted resistance values of the pure Sn/C and Sn@C/SiOC electrodes upon cycling measured using a simplified equivalent circuit and Z-view program.

| Simplified equivalent circuit | | 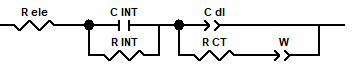 | | |
| --- | --- | --- | --- | --- |
| Electrode | Component | Resistance (Ω) | | |
|  |  | Before cycle | After 30 cycles | After 400 cycles |
| Pure Sn/C | *R_ele_* | 1.51 | 2.03 | 2.11 |
|  | *R_int_* | - | 17.55 | 39.08 |
|  | *R_ct_* | 536.4 | 19.25 | 179.2 |
| Sn@C/SiOC | *R_ele_* | 1.75 | 2.64 | 3.25 |
|  | *R_int_* | - | 1.25 | 1.66 |
|  | *R_ct_* | 440.5 | 1.82 | 3.00 |

**Table S3.** Impedance parameters and diffusion coefficient values of the pure Sn/C and Sn@C/SiOC electrodes during the 1^st^ (de)sodiation processes at various voltages.

| Materials | | Parameters | Measured and calculated values | | | |
| --- | --- | --- | --- | --- | --- | --- |
| Pure Sn/C | Step | | 1^st^ Sodiation | | | |
|  | Voltage (V) | | 0.8 | 0.5 | 0.1 | 0.001 |
|  | *R_tot_* (Ω) | | 517 | 455 | 28 | 16.5 |
|  | *D_Na+_* (cm^2^ s^-1^) | | 1.56E-14 | 2.66E-11 | 3.99E-10 | 2.84E-09 |
|  | Step | | 1^st^ Desodiation | | | |
|  | Voltage (V) | | 0.1 | 0.3 | 0.5 | 0.8 |
|  | *R_tot_* (Ω) | | 15.4 | 7.3 | 6.9 | 5.3 |
|  | *D_Na+_* (cm^2^ s^-1^) | | 9.98E-09 | 6.87E-10 | 1.51E-10 | 8.76E-12 |
| Sn@C/SiOC nanohybrid | Step | | 1^st^ Sodiation | | | |
|  | Voltage (V) | | 0.8 | 0.5 | 0.1 | 0.001 |
|  | *R_tot_* (Ω) | | 346 | 186.2 | 12.8 | 6.6 |
|  | *D_Na+_* (cm^2^ s^-1^) | | 8.76E-14 | 3.04E-11 | 1.32E-09 | 7.89E-09 |
|  | Step | | 1^st^ Desodiation | | | |
|  | Voltage (V) | | 0.1 | 0.3 | 0.5 | 0.8 |
|  | *R_tot_* (Ω) | | 5.7 | 3.9 | 3.7 | 4 |
|  | *D_Na+_* (cm^2^ s^-1^) | | 6.39E-09 | 9.10E-10 | 2.89E-10 | 5.14E-11 |

**Table S4.** Impedance parameters and diffusion coefficient values of pure Sn/C and Sn@C/SiOC electrodes during the 2^nd^ (de)sodiation processes at various voltages.

| Materials | | Parameters | Measured and calculated values | | | |
| --- | --- | --- | --- | --- | --- | --- |
| Pure Sn/C | Step | | 2^nd^ Sodiation | | | |
|  | Voltage (V) | | 0.8 | 0.5 | 0.1 | 0.001 |
|  | *R_tot_* (Ω) | | 5.3 | 9 | 20 | 8.8 |
|  | *D_Na+_* (cm^2^ s^-1^) | | 8.76E-12 | 8.84E-11 | 6.87E-10 | 4.09E-09 |
|  | Step | | 2^nd^ Desodiation | | | |
|  | Voltage (V) | | 0.1 | 0.3 | 0.5 | 0.8 |
|  | *R_tot_* (Ω) | | 7.6 | 4.2 | 4.2 | 3.7 |
|  | *D_Na+_* (cm^2^ s^-1^) | | 3.78E-09 | 5.07E-10 | 7.72E-11 | 1.02E-11 |
| Sn@C/SiOC nanohybrid | Step | | 2^nd^ Sodiation | | | |
|  | Voltage (V) | | 0.8 | 0.5 | 0.1 | 0.001 |
|  | *R_tot_* (Ω) | | 4 | 4.2 | 5 | 5.4 |
|  | *D_Na+_* (cm^2^ s^-1^) | | 5.14E-11 | 2.51E-10 | 3.78E-09 | 8.84E-09 |
|  | Step | | 2^nd^ Desodiation | | | |
|  | Voltage (V) | | 0.1 | 0.3 | 0.5 | 0.8 |
|  | *R_tot_* (Ω) | | 4.9 | 4.1 | 4.2 | 3.9 |
|  | *D_Na+_* (cm^2^ s^-1^) | | 5.80E-09 | 8.45E-10 | 2.56E-10 | 5.28E-11 |

| Active material | Initial sodiation capacity  [mAh g^-1^ / rate] | Initial desodiation capacity  [mAh g^-1^ / rate] | ICE [%] | Cycling stability  [mAh g^-1^ /@cycle #  / retention / rate] | Rate capability  [mAh g^-1^ / rate] | Loading mass  [mg/cm^2^] | Refs. |
| --- | --- | --- | --- | --- | --- | --- | --- |
| Sn/NMC | 936 / 0.05 A g^-1^ | 439 / 0.05 A g^-1^ | 46.9 | 332 / 300 / 75.6% / 0.05 A g^-1^ | 149 / 5 A g^-1^ | 0.8 | [60] |
| Sn/SnO@C | 1243 / 0.1 A g^-1^ | 544 / 0.1 A g^-1^ | 45 | 401 / 100 / 73.7% / 0.1 A g^-1^ | 130 / 2 A g^-1^ | 1.0-1.2 | [61] |
| Sn@C@CNF | 690.3 / 0.1 A g^-1^ | 485.1 / 0.1 A g^-1^ | 70.3 | 360.5 / 100 / 74.3% / 0.1 A g^-1^ | 212.2 / 1 A g^-1^ | 2.0 | [62] |
| Sn/C | ~900 / 0.1 A g^-1^ | ~550 / 0.1 A g^-1^ | 61.1 | 334.8 / 1,000 / 65.5% / 0.5 A g^-1^ | 250 / 5 A g^-1^ | 1.4-1.8 | [63] |
| C/SnO_2_/Sn@C | 958.3 / 0.1 A g^-1^ | 750.8 / 0.1 A g^-1^ | 78.4 | 247.9 / 50 / - / 0.2 A g^-1^ | - | 1.1 | [64] |
| Sn/Sn_4_P_3_ @MXene | 693.3 / 0.1 A g^-1^ | 455.5 / 0.1 A g^-1^ | 65.7 | ~100 / 1,000 / - / 5 A g^-1^ | 165.4 / 5 A g^-1^ | 1.0 | [65] |
| Sn@C/SiOC | **624.6 / 0.05 A g^-1^** | **504.0 / 0.05 A g^-1^** | **80.7** | **421.1 / 1,500 / 91.9% / 5 A g^-1^** | **420.2 / 10 A g^-1^** | **1.5-4.0** | **This work** |

**Table S5.** Summary of sodium storage performance of various Sn-based anodes for SIBs.
